# Supplementary material for: Nucleolar- and Nuclear-Stress-Induced Membrane-Less Organelles: A Proteome Analysis through the Prism of Liquid–Liquid Phase Separation
Source: Int J Mol Sci. 2023 Jul 2;24(13):11007. doi: 10.3390/ijms241311007 (PMC10341607; doi:10.3390/ijms241311007)
Supplement: Supplementary file 1 [file ijms-24-11007-s001.zip › ijms-2400797-supplementary.pdf]

Supplementary Materials

Nucleolar and nuclear stress-induced membrane-less organelles: A proteome analysis through the prism of liquid-liquid phase separation

Yakov I. Mokin [1], Anastasia A. Gavrilova [1], Anna S. Fefilova [1,†], Irina M. Kuznetsova [1], Konstantin K. Turoverov [1], Vladimir N. Uversky [2,*] and Alexander V. Fonin [1,*]

[1] Laboratory of structural dynamics, stability and folding of proteins, Institute of Cytology, Russian Academy of Sciences, St. Petersburg 194064, Russia; mokinyakov@mail.ru (Y.I.M.); asultanbekova@incras.ru (A.A.G.); anny.fefilova@gmail.com (A.S.F.); imk@incras.ru (I.M.K.); kkt@incras.ru (K.K.T.); alexfonin@incras.ru (A.V.F.)

[2] Department of Molecular Medicine and USF Health Byrd Alzheimer's Research Institute, Morsani College of Medicine, University of South Florida, Tampa, FL 33612, USA; vuversky@usf.edu

† Current address: Center of Genomic Regulation (GRC), Barcelona Institute of Science and Technology, Barcelona, 08003, Spain.

* Correspondence: alexfonin@incras.ru (A.V.F.) and vuversky@usf.edu (V.N.U.)

Supplementary **Figures S1-S10** represent amino acid sequences and funcitonal disorder profiles of 10 human proteins shared by the acidosis-induced A-bodies, heat-induced A-bodies, and nSBs. In each figure, the upper plot represents amino acid sequence in FASTA format, whereas the bottom plot shows corresponding functional profile generated by the D²P² platform. In these profiles, the IDPR localization predicted by IUPred, PONDR® VLXT, PONDR® VSL2, PrDOS, PV2, and ESpritz are shown by 9 differently colored bars on the top of the plot, whereas the blue-green-white bar in the middle of the plots shows the agreement between the outputs of these disorder predictors, with disordered regions by consensus being shown by blue and green. The two lines with colored and numbered bars above the disorder consensus bar show the positions of functional SCOP domains [40, 41] predicted using the SUPERFAMILY predictor [42]. Positions of the predicted disorder-based binding sites (molecular recognition feature (MoRFs) identified by the ANCHOR algorithm are shown by yellow zigzagged bars [43]. Locations of the sites of different posttranslational modifications (PTMs) identified by the PhosphoSitePlus platform [44] are shown at the bottom of the plots by the differently colored circles.

Figure S1. Amino acid sequence and functional disorder profile of Nucleolin (NCL; also known as Protein C23; UniProt ID: P19338).

```
>sp|P19338|NUCL_HUMAN Nucleolin OS=Homo sapiens OX=9606 GN=NCL PE=1 SV=3
MVKLAKAGKNQGDPKKMAPPPKEVEEDSEDEEMSEDEEDDSSGEEVVIPQKKGKKAAATSAKKVVVSPTKKVAVATP
AKKAAVTPGKKAAATPAKKTVTPAKAVTTPGKKGATPGKALVATPGKKGAAIPAKGAKNGKNAKKEDSDEEEDDDSE
EDEEDDEDEDEDEDEIEPAAMKAAAAAPASEDEDDEDDEDDEDDDDDEEDDSEEEAMETTPAKGKKAAKVVPVKAKN
VAEDEDEEEDDEDEDDDDDEDDEDDDDEDDEEEEEEEEEEPVKEAPGKRKKEMAKQKAAPEAKKQKVEGTEPTTAFN
LFVGNLNFNKSAPELKTGISDVFAKNDLAVVDVRIGMTRKFGYVDFESAEDLEKALELTGLKVFGNEIKLEKPKGKD
SKKERDARTLLAKNLPYKVTQDELKEVFEDAAEIRLVSKDGKSKGIAYIEFKTEADAEKTFEEKQGTEIDGRSISLY
YTGEKGQNQDYRGGKNSTWSGESKTLVLSNLSYSATEETLQEVFEKATFIKVPQNQNGKSKGYAFIEFASFEDAKEA
LNSCNKREIEGRAIRLELQGPRGSPNARSQPSKTLFVKGLSEDTTEETLKESFDGSVRARIVTDRETGSSKGFGFVD
FNSEEDAKAAKEAMEDGEIDGNKVTLDWAKPKGEGGFGGRGGGRGGFGGRGGGRGGRGGFGGRGRGGFGGRGGFRGG
RGGGGDHKPQGKKTKFE
```

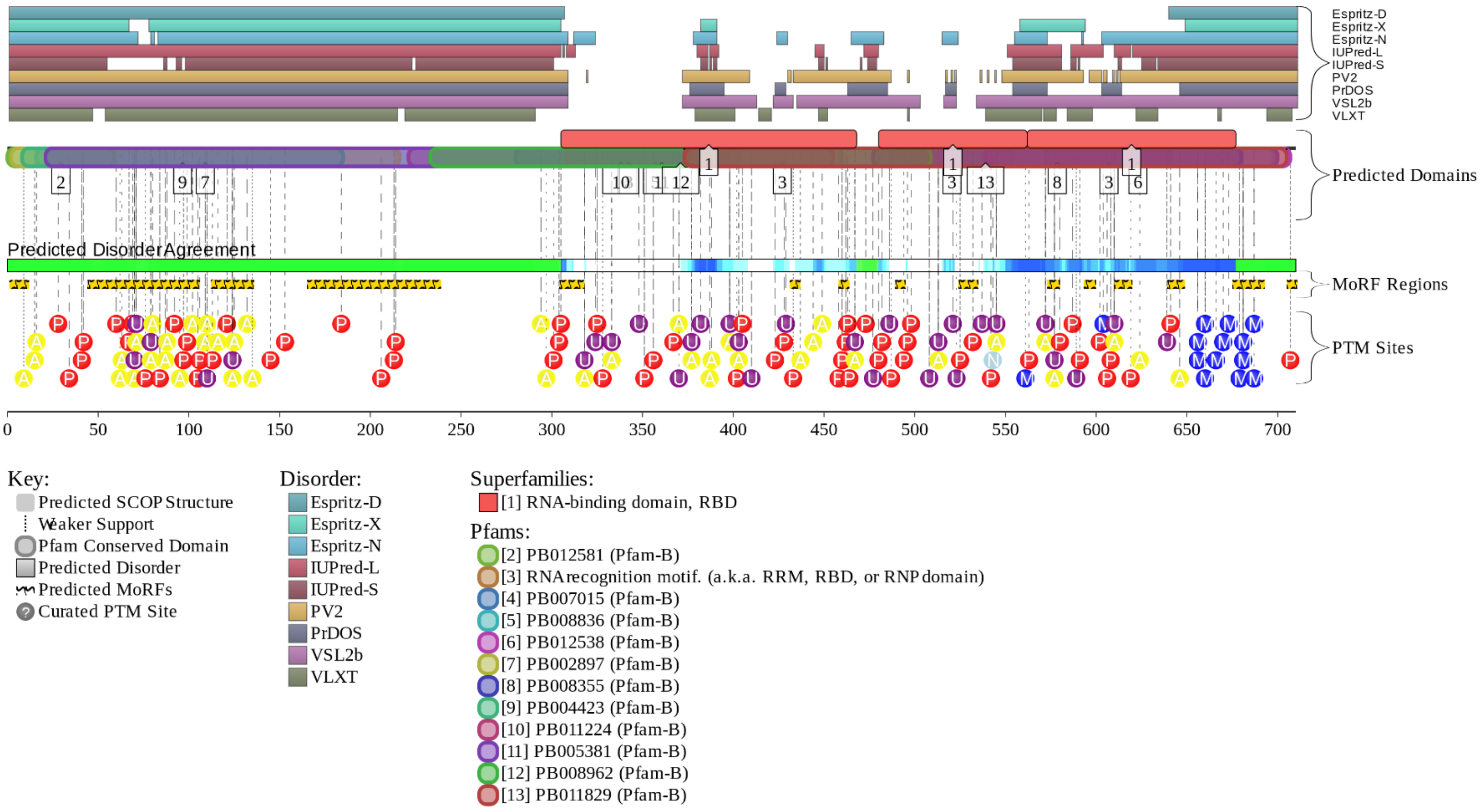

Figure S2. Amino acid sequence and functional disorder profile of heterogeneous nuclear ribonucleoprotein M (HNRNPM; UniProt ID: P52272).

```
>sp|P52272|HNRPM_HUMAN Heterogeneous nuclear ribonucleoprotein M OS=Homo
sapiens OX=9606 GN=HNRNPM PE=1 SV=3
MAAGVEAAAEVAATEIKMEEESGAPGVPSGNGAPGPKGEGERPAQNEKRKEKNIKRGGNRFEPYANPTKRYRAFITN
IPFDVKWQSLKDLVKEKVGEVTYVELLMDAEGKSRGCAVVEFKMEESMKKAAEVLNKHSLSGRPLKVKEDPDGEHAR
RAMQKVMATTGGMGMGPGGPGMITIPPSILNNPNIPNEIIHALQAGRLGSTVFVANLDYKVGWKKLKEVFSMAGVVV
RADILEDKDGKSRGIGTVTFEQSIEAVQAISMFNGQLLFDRPMHVKMDERALPKGDFFPPERPQQLPHGLGGIGMGL
GPGGQPIDANHLNKGIGMGNIGPAGMGMEGIGFGINKMGGMEGPFGGGMENMGRFGSGMNMGRINEILSNALKRGEI
IAKQGGGGGGGSVPGIERMGPGIDRLGGAGMERMGAGLGHGMDRVGSEIERMGLVMDRMGSVERMGSGIERMGPLGL
DHMASSIERMGQTMERIGSGVERMGAGMGFGLERMAAPIDRVGQTIERMGSGVERMGPAIERMGLSMERMVPAGMGA
GLERMGPVMDRMATGLERMGANNLERMGLERMGANSLERMGLERMGANSLERMGPAMGPALGAGIERMGLAMGGGGG
ASFDRAIEMERGNFGGSFAGSFGGAGGHAPGVARKACQIFVRNLPFDFTWKMLKDKFNECGHVLYADIKMENGKSKG
CGVVKFESPEVAERACRMMNGMKLSGREIDVRIDRNA
```

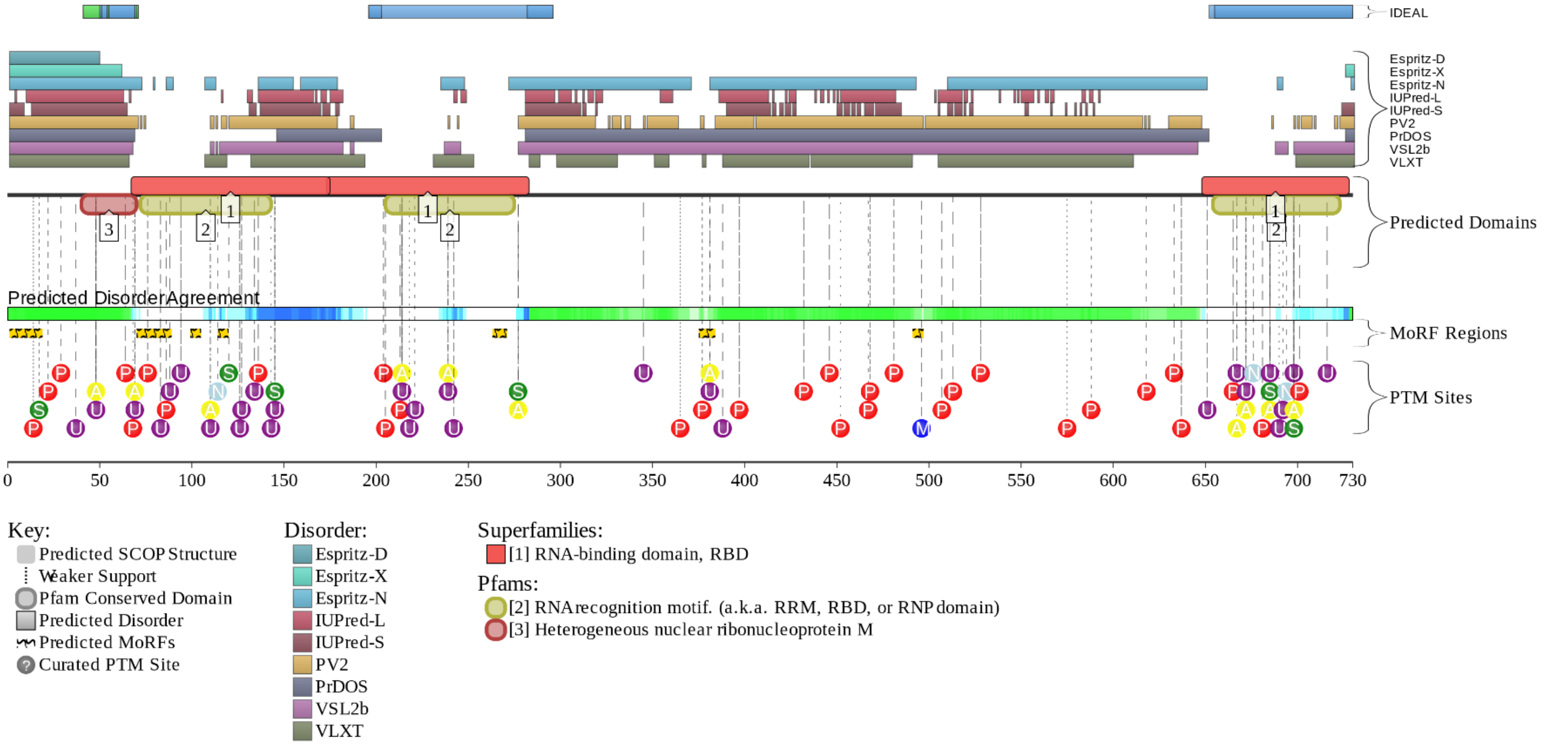

Figure S3. Amino acid sequence and functional disorder profile of putative RNA-binding protein Luc7-like 2 (LUC7L2, also known as CGI-74; UniProt ID: Q9Y383).

```
>sp|Q9Y383|LC7L2_HUMAN Putative RNA-binding protein Luc7-like 2 OS=Homo
sapiens OX=9606 GN=LUC7L2 PE=1 SV=2
MSAQAQMRAMLDQLMGTSRDGDTTRQRIKFSDDRVCKSHLLNCCPHDVLSGTRMDLGECLKVHDLALRADYEIASKE
QDFFFELDAMDHLQSFIADCDRRTEVAKKRLAETQEEISAEVAAKAERVHELNEEIGKLLAKVEQLGAEGNVEESQK
VMDEVEKARAKKREAEEVYRNSMPASSFQQQKLRVCEVCSAYLGLHDNDRRLADHFGGKLHLGFIEIREKLEELKRV
VAEKQEKRNQERLKRREEREREEREKLRRSRSHSKNPKRSRSREHRRHRSRSMSRERKRRTRSKSREKRHRHRSRSS
SRSRSRSHQRSRHSSRDRSRERSKRRSSKERFRDQDLASCDRDRSSRDRSPRDRDRKDKKRSYESANGRSEDRRSSE
EREAGEI
```

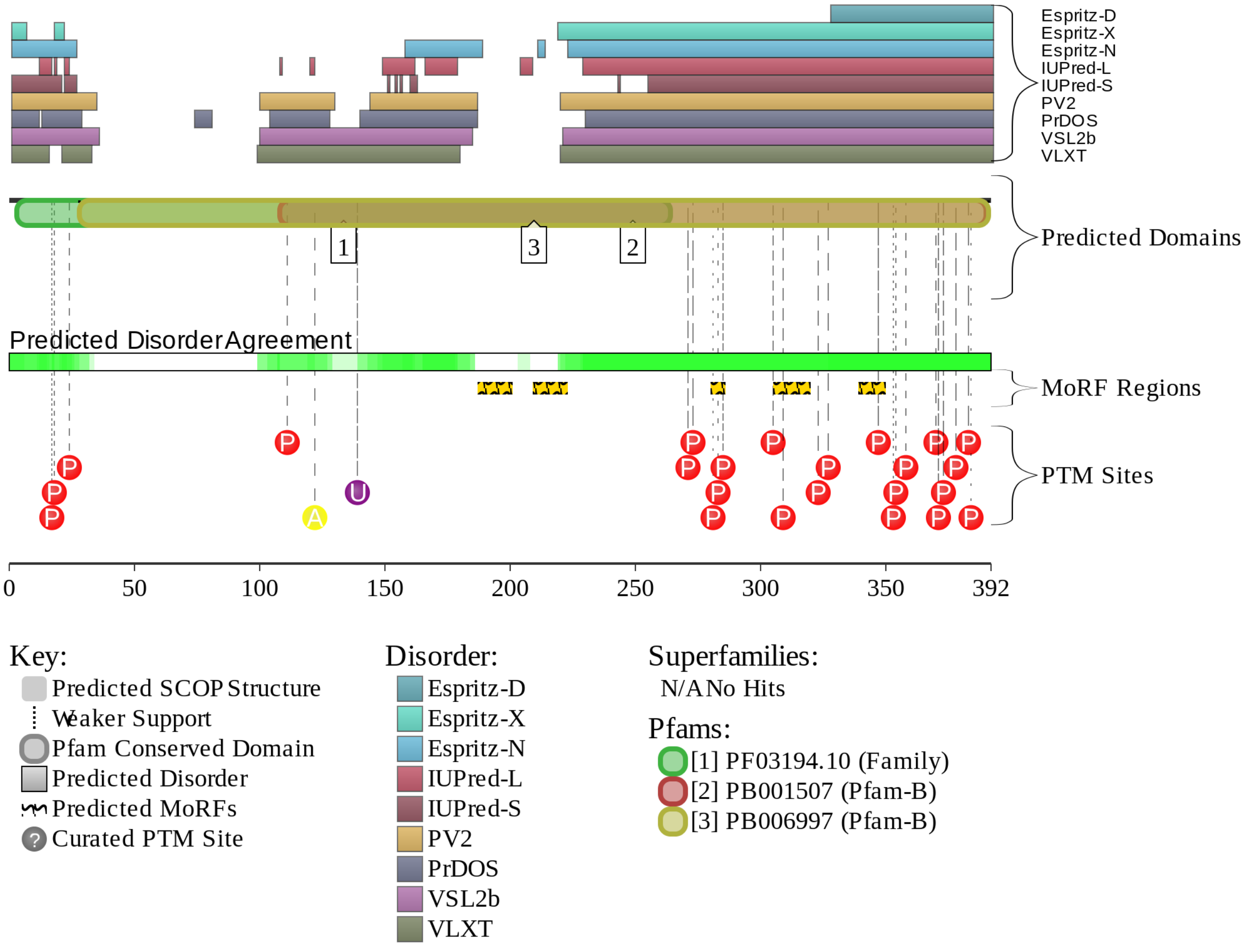

Figure S4. Amino acid sequence and functional disorder profile of heterogeneous nuclear ribonucleoprotein A0 (HNRNPA0; UniProt ID: Q13151).

```
>sp|Q13151|ROA0_HUMAN Heterogeneous nuclear ribonucleoprotein A0 OS=Homo
sapiens OX=9606 GN=HNRNPA0 PE=1 SV=1
MENSQLCKLFIGGLNVQTSESGLRGHFEAFGTLTDCVVVVNPQTKRSRCFGFVTYSNVEEADAAMAASPHAVDGNTV
ELKRAVSREDSARPGAHAKVKKLFVGGLKGDVAEGDLIEHFSQFGTVEKAEIIADKQSGKKRGFGFVYFQNHDAADK
AAVVKFHPIQGHRVEVKKAVPKEDIYSGGGGGGSRSSRGGRGGRGRGGGRDQNGLSKGGGGGYNSYGGYGGGGGGGY
NAYGGGGGGSSYGGSDYGNGFGGFGSYSQHQSSYGPMKSGGGGGGGGSSWGGRSNSGPYRGGYGGGGGYGGSSF
```

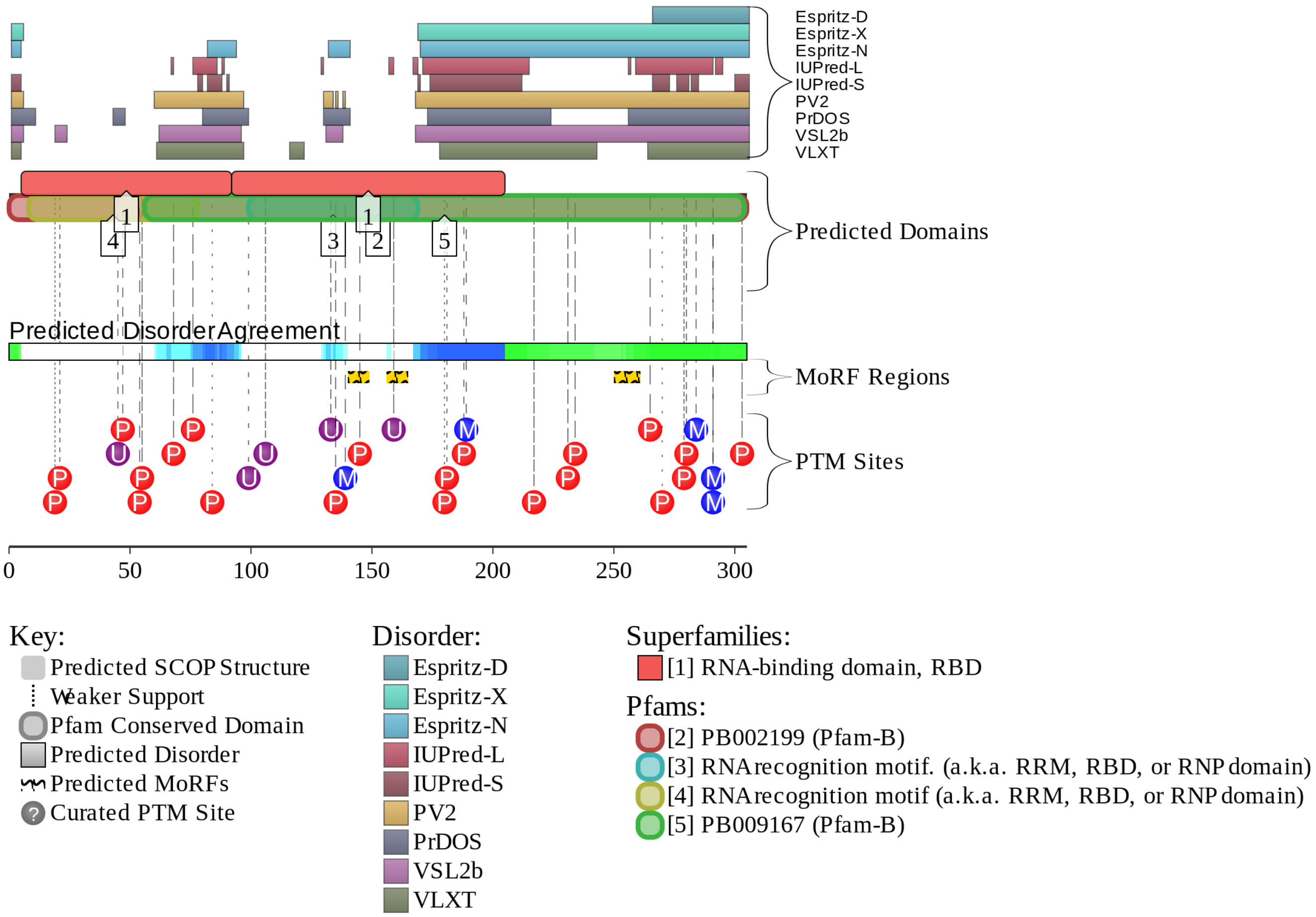

Figure S5. Amino acid sequence and functional disorder profile of heterogeneous nuclear ribonucleoprotein K (HNRNPK; UniProt ID: P61978).

```
>sp|P61978|HNRPK_HUMAN Heterogeneous nuclear ribonucleoprotein K OS=Homo
sapiens OX=9606 GN=HNRNPK PE=1 SV=1
METEQPEETFPNTETNGEFGKRPAEDMEEEQAFKRSRNTDEMVELRILLQSKNAGAVIGKGGKNIKALRTDYNASVS
VPDSSGPERILSISADIETIGEILKKIIPTLEEGLQLPSPTATSQLPLESDAVECLNYQHYKGSDFDCELRLLIHQS
LAGGIIGVKGAKIKELRENTQTTIKLFQECCPHSTDRVVLIGGKPDRVVECIKIILDLISESPIKGRAQPYDPNFYD
ETYDYGGFTMMFDDRRGRPVGFPMRGRGGFDRMPPGRGGRPMPPSRRDYDDMSPRRGPPPPPPGRGGRGGSRARNLP
LPPPPPPRGGDLMAYDRRGRPGDRYDGMVGFSADETWDSAIDTWSPSEWQMAYEPQGGSGYDYSYAGGRGSYGDLGG
PIITTQVTIPKDLAGSIIGKGGQRIKQIRHESGASIKIDEPLEGSEDRIITITGTQDQIQNAQYLLQNSVKQYSGKF
F
```

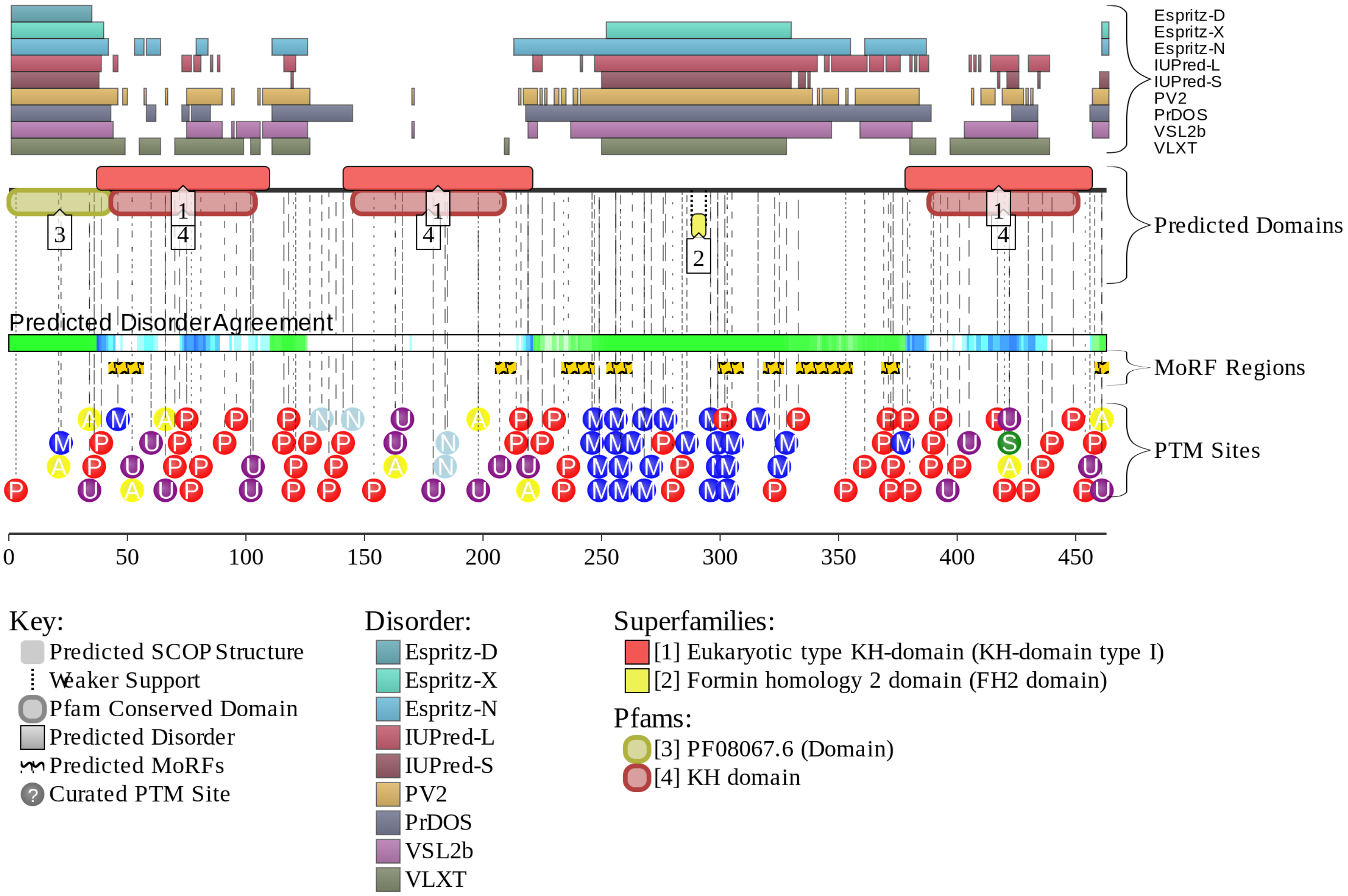

Figure S6. Amino acid sequence and functional disorder profile of RNA-binding protein 39 (RBM39; also known as CAPER alpha; Hepatocellular carcinoma protein 1; RNA-binding motif protein 39; RNA-binding region-containing protein 2; and Splicing factor HCC1UniProt ID: Q14498).

```
>sp|Q14498|RBM39_HUMAN RNA-binding protein 39 OS=Homo sapiens OX=9606
GN=RBM39 PE=1 SV=2
MADDIDIEAMLEAPYKKDENKLSSANGHEERSKKRKKSKSRSRSHERKRRSKSKERKRSRDRERKKSKSRERKRSRSK
ERRRSRSRSRDRRFRGRYRSPYSGPKFNSAIRGKIGLPHSIKLSRRRSRSKSPFRKDKSPVREPIDNLTPEERDART
VFCMQLAARIRPRDLEEFFSTVGKVRDVRMISDRNSRRSKGIAYVEFVDVSSVPLAIGLTGQRVLGVPIIVQASQAE
KNRAAAMANNLQKGSAGPMRLYVGSLHFNITEDMLRGIFEPFGRIESIQLMMDSETGRSKGYGFITFSDSECAKKAL
EQLNGFELAGRPMKVGHVTERTDASSASSFLDSDELERTGIDLGTTGRLQLMARLAEGTGLQIPPAAQQALQMSGSL
AFGAVAEFSFVIDLQTRLSQQTEASALAAAASVQPLATQCFQLSNMFNPQTEEEVGWDTEIKDDVIEECNKHGGVIH
IYVDKNSAQGNVYVKCPSIAAAIAAVNALHGRWFAGKMITAAYVPLPTYHNLFPDSMTATQLLVPSRR
```

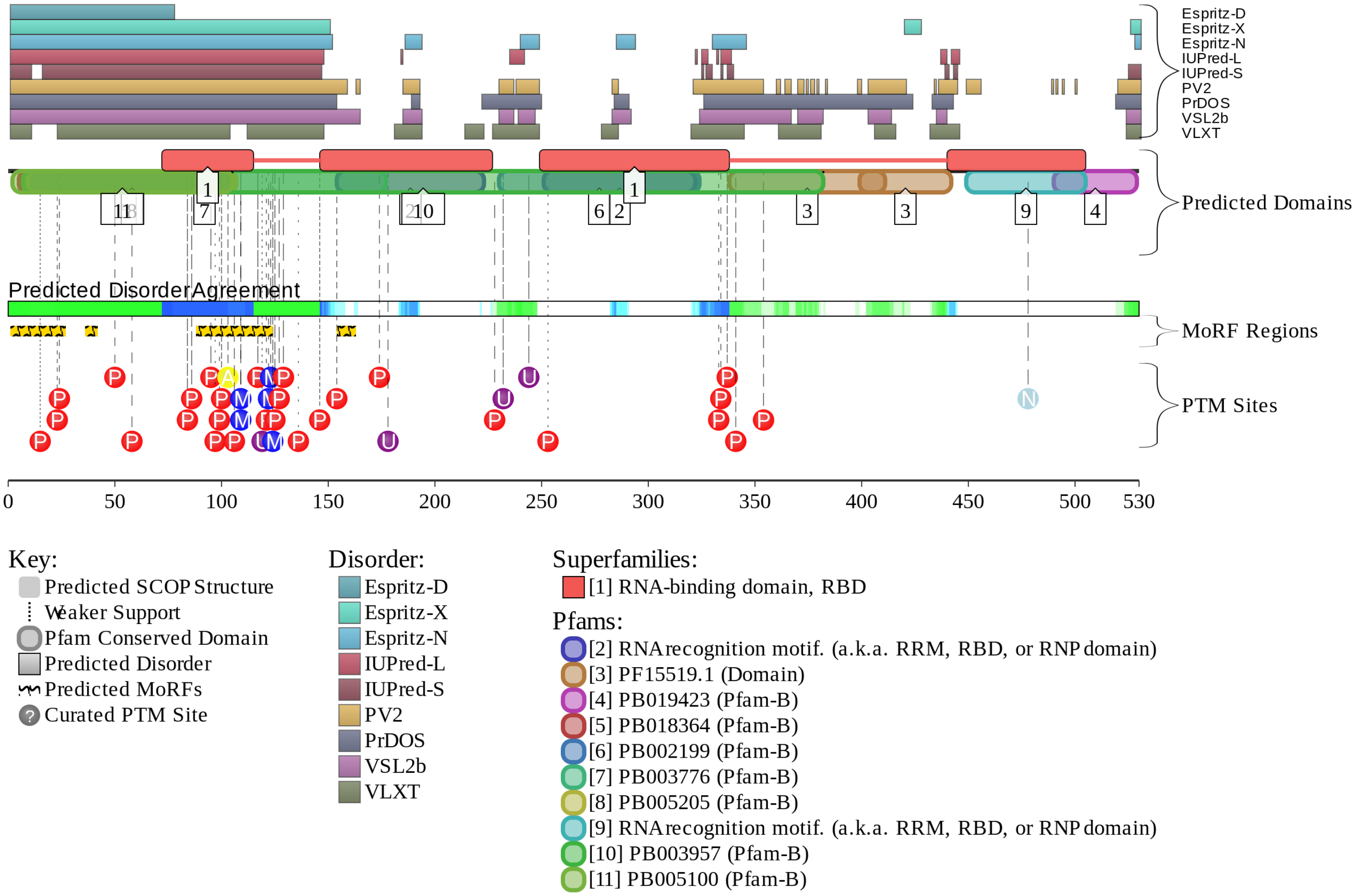

Figure S7. Amino acid sequence and functional disorder profile of heterogeneous nuclear ribonucleoprotein H (HNRNPH1; UniProt ID: P31943).

```
>sp|P31943|HNRH1_HUMAN Heterogeneous nuclear ribonucleoprotein H OS=Homo
sapiens OX=9606 GN=HNRNPH1 PE=1 SV=4
MMLGTEGGEGFVVKVRGLPWSCSADEVQRFFSDCKIQNGAQGIRFIYTREGRPSGEAFVELESEDEVKLALKKDRET
MGHRYVEVFKSNNVEMDWVLKHTGPNSPDTANDGFVRLRGLPFGCSKEEIVQFFSGLEIVPNGITLPVDFQGRSTGE
AFVQFASQEIAEKALKKHKERIGHRYIEIFKSSRAEVRTHYDPPRKLMAMQRPGPYDRPGAGRGYNSIGRGAGFERM
RRGAYGGGYGGYDDYNGYNDGYGFGSDRFGRDLNYCFSGMSDHRYGDGGSTFQSTTGHCVHMRGLPYRATENDIYNF
FSPLNPVRVHIEIGPDGRVTGEADVEFATHEDAVAAMSKDKANMQHRYVELFLNSTAGASGGAYEHRYVELFLNSTA
GASGGAYGSQMMGGMGLSNQSSYGGPASQQLSGGYGGGYGGQSSMSGYDQVLQENSSDFQSNIA
```

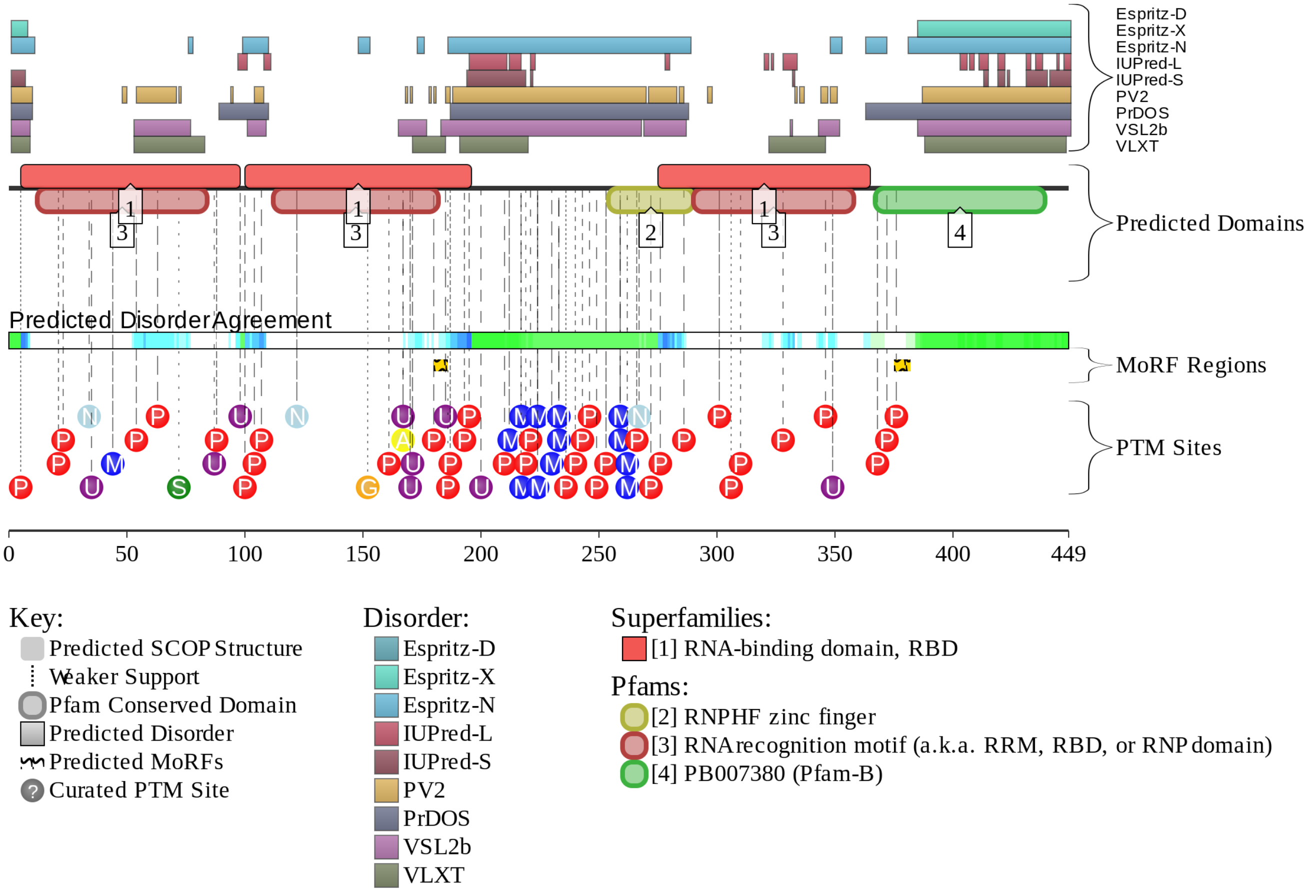

Figure S8. Amino acid sequence and functional disorder profile of heat shock cognate 71 kDa protein (also known as heat shock 70 kDa protein 8 (HSPA8) and Lipopolysaccharide-associated protein 1 (LAP-1; LPS-associated protein 1); UniProt ID: P11142).

```
>sp|P11142|HSP7C_HUMAN Heat shock cognate 71 kDa protein OS=Homo sapiens
OX=9606 GN=HSPA8 PE=1 SV=1
MSKGPAVGIDLGTTYSCVGVFQHGKVEIIANDQGNRTTPSYVAFTDTERLIGDAAKNQVAMNPTNTVFDAKRLIGRR
FDDAVVQSDMKHWPFMVVNDAGRPKVQVEYKGETKSFYPEEVSSMVLTKMKEIAEAYLGKTVTNAVVTVPAYFNDSQ
RQATKDAGTIAGLNVLRIINEPTAAAIAYGLDKKVGAERNVLIFDLGGGTFDVSILTIEDGIFEVKSTAGDTHLGGE
DFDNRMVNHFIAEFKRKHKKDISENKRAVRRLRTACERAKRTLSSSTQASIEIDSLYEGIDFYTSITRARFEELNAD
LFRGTLDPVEKALRDAKLDKSQIHDIVLVGGSTRIPKIQKLLQDFFNGKELNKSINPDEAVAYGAAVQAAILSGDKS
ENVQDLLLLDVTPLSLGIETAGGVMTVLIKRNTTIPTKQTQTFTTYSDNQPGVLIQVYEGERAMTKDNNLLGKFELT
GIPPAPRGVPQIEVTFDIDANGILNVSAVDKSTGKENKITITNDKGRLSKEDIERMVQEAEKYKAEDEKQRDKVSSK
NSLESYAFNMKATVEDEKLQGKINDEDKQKILDKCNEIINWLDKNQTAEKEEFEHQQKELEKVCNPIITKLYQSAGG
MPGGMPGGFPGGGAPPSGGASSGPTIEEVD
```

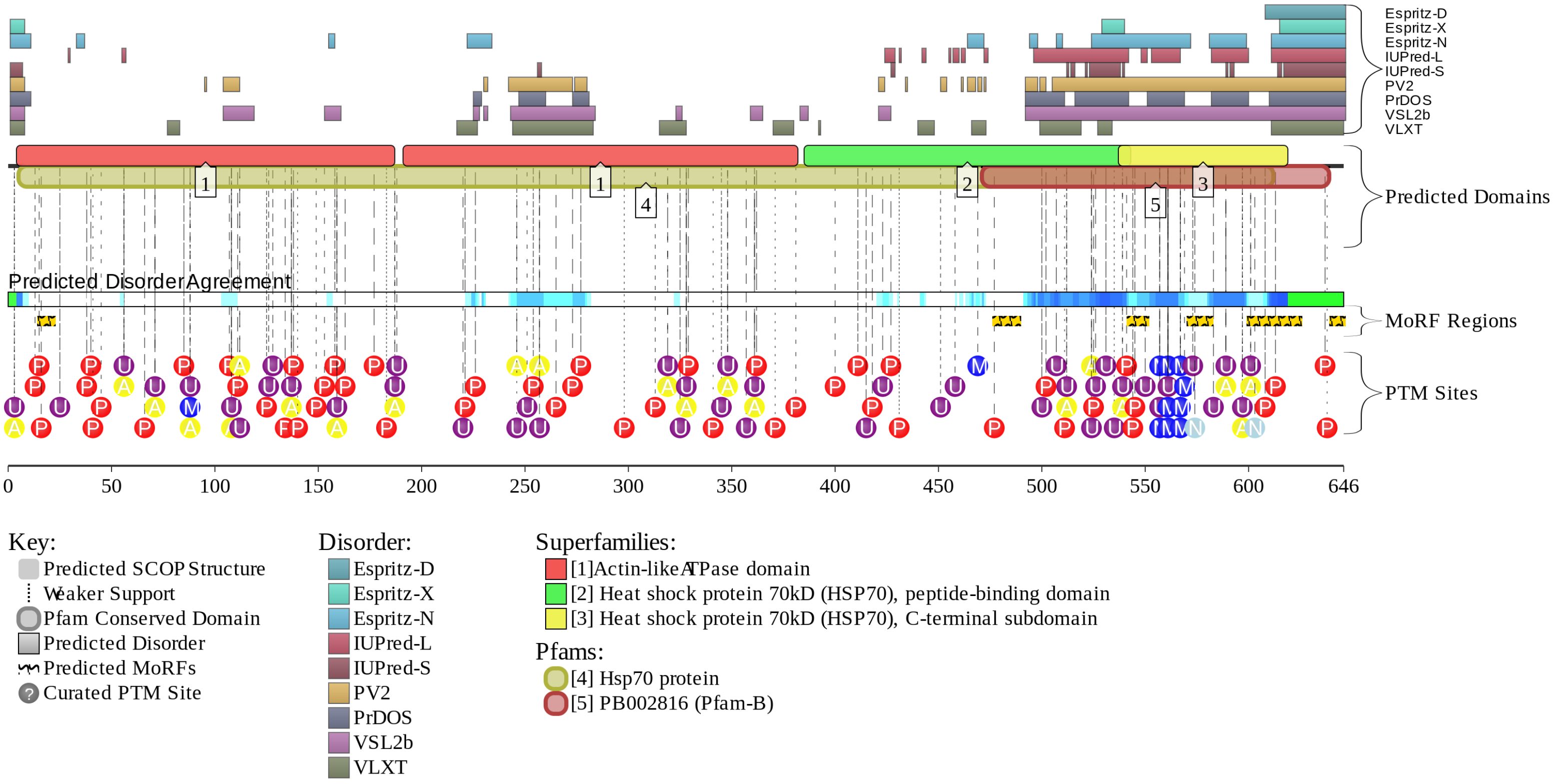

Figure S9. Amino acid sequence and functional disorder profile of peptidyl-prolyl cis-trans isomerase A (PPIA, UniProt ID: P62937).

```
>sp|P62937|PPIA_HUMAN Peptidyl-prolyl cis-trans isomerase A OS=Homo sapiens
OX=9606 GN=PPIA PE=1 SV=2
MVNPTVFFDIAVDGEPLGRVSFELFADKVPKTAENFRALSTGEKGFGYKGSCFHRIIPGFMCQGGDFTRHNGTGGKS
IYGEKFEDENFILKHTGPGILSMANAGPNTNGSQFFICTAKTEWLDGKHVVFGKVKEGMNIVEAMERFGSRNGKTSK
KITIADCGQLE
```

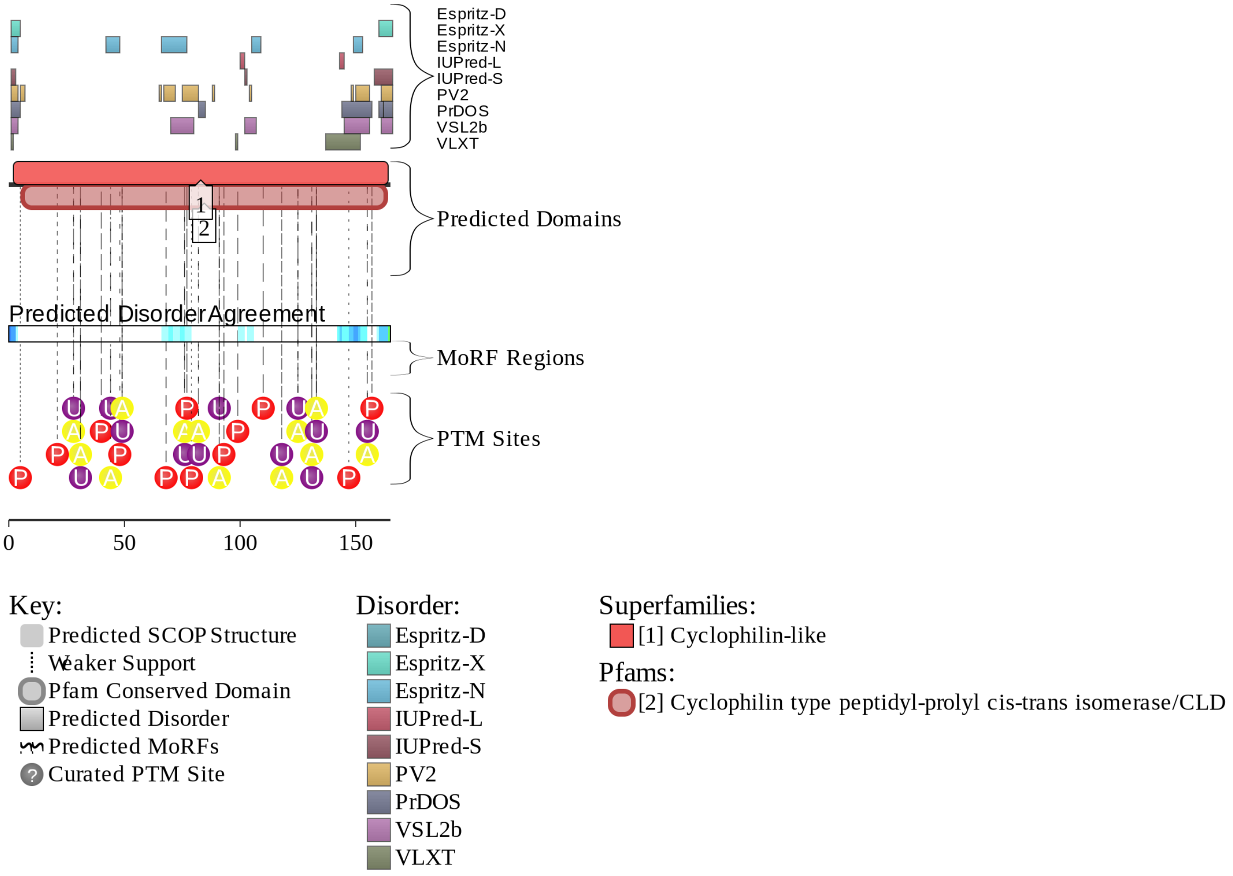

Figure S10. Amino acid sequence and functional disorder profile of ATP-dependent RNA helicase DDX39A (also known as DEAD box protein 39 (DDX39A) and Nuclear RNA helicase URH49 (UniProt ID: O00148).

```
>sp|O00148|DX39A_HUMAN ATP-dependent RNA helicase DDX39A OS=Homo sapiens
OX=9606 GN=DDX39A PE=1 SV=2
MAEQDVENDLLDYDEEEEPQAPQESTPAPPKKDIKGSYVSIHSSGFRDFLLKPELLRAIVDCGFEHPSEVQHECIPQ
AILGMDVLCQAKSGMGKTAVFVLATLQQIEPVNGQVTVLVMCHTRELAFQISKEYERFSKYMPSVKVSVFFGGLSIK
KDEEVLKKNCPHVVVGTPGRILALVRNRSFSLKNVKHFVLDECDKMLEQLDMRRDVQEIFRLTPHEKQCMMFSATLS
KDIRPVCRKFMQDPMEVFVDDETKLTLHGLQQYYVKLKDSEKNRKLFDLLDVLEFNQVIIFVKSVQRCMALAQLLVE
QNFPAIAIHRGMAQEERLSRYQQFKDFQRRILVATNLFGRGMDIERVNIVFNYDMPEDSDTYLHRVARAGRFGTKGL
AITFVSDENDAKILNDVQDRFEVNVAELPEEIDISTYIEQSR
```

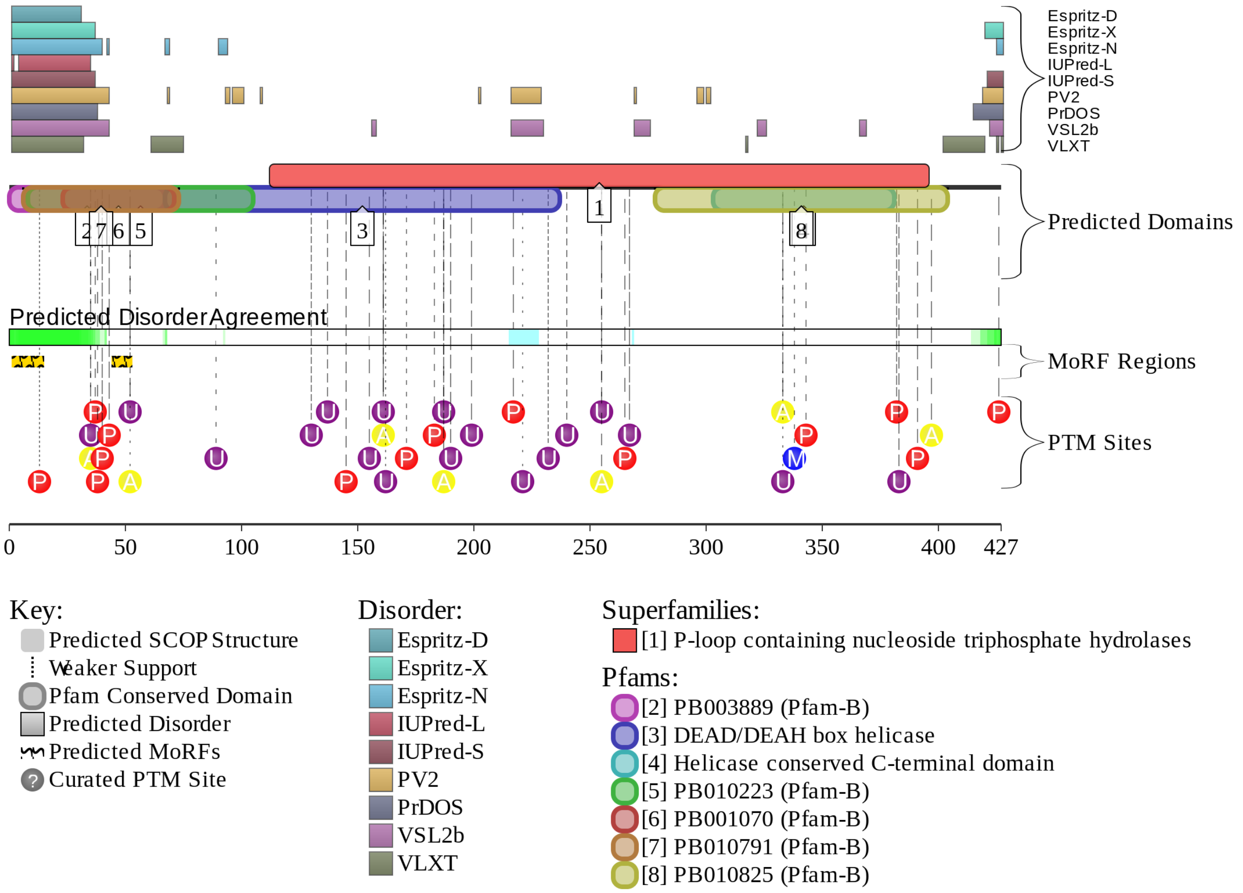

Table S1. Characteristics of the proteome of the heat shock-induced A-bodies. DPR are droplet-promoting regions, nHS are number of aggregation hot spots, Na4vSS are Normalized a4v Sequence Sum for 100 residues used for aggregation prediction.

Gene Name	UniProtID	Fasta	Sequence	PER(VSL2B)	Disorder Classifications	FuzDrop Score	FuzDrop LLPS	PSPredictor Score	PSPredictor LLPS	Fuz&PSP LLPS	Fuz&PSP No LLPS	Number of DPRs	Role in MLO	nHS	Na4vSS	Agregation Predicted	Aggregation DB scanning	Hydrophobicity	Charge
VHL	P40337	>sp|P4033	PGTGRRIH	55.87	IDP	0.6579	Yes	0.0102	No			2	Driver	6	-23	No	Yes	-0.78	-0.073
GSTM3	P21266	>sp|P2126	MSCESSM	14.22	Partial IDP	0.1175	No	0.0047	No		+	0	No	9	-2.8	No	No	-0.41	-0.016
FEN1	P39748	>sp|P3974	MGIQGLA	44.47	IDP	0.2197	No	0.2899	No		+	2	Client	11	-14.1	No	No	-0.53	0.026
MPP10	O00566	>sp|O005	MAPQVW	76.95	IDP	0.9394	Yes	0.2793	No			7	Driver	9	-36.6	No	No	-1.11	-0.070
PCBP2	Q15366	>sp|Q153	MDTGVIE	44.93	IDP	0.9028	Yes	0.2466	No			3	Driver	10	-3.4	No	No	-0.14	0.003
AN32B	Q92688	>sp|Q926	MDMKRRI	61.75	IDP	0.4995	No	0.5683	Yes			1	Client	3	-47.7	No	No	-1.16	-0.263
MYL6	P60660	>sp|P6066	MCDFTED	19.21	Partial IDP	0.1325	No	0.0072	No		+	0	No	7	-13.2	No	No	-0.39	-0.079
LC7L2	Q9Y383	>sp|Q9Y3	MSAQAQN	74.49	IDP	0.9512	Yes	0.0666	No			3	Driver	2	-50.2	No	No	-1.49	0.088
RRP12	Q5JTH9	>sp|Q5JTH	MGRSGKL	36.24	IDP	0.5879	No	0.5229	Yes			7	Client	44	-4.9	No	No	-0.23	0.032
COMT	P21964	>sp|P2196	MPEAPPLL	7.01	Ordered	0.1043	No	0.0046	No		+	0	No	9	9.1	Yes	No	0.16	-0.022
POP1	Q99575	>sp|Q995	MSNAKER	50.88	IDP	0.9448	Yes	0.6756	Yes	+		8	Driver	37	-16	No	No	-0.62	0.056
UT14A	Q9BVJ6	>sp|Q9BV	MTANRLA	81.06	IDP	0.9196	Yes	0.3773	No			6	Driver	5	-29.4	No	No	-0.94	0.011
HSPB1	P04792	>sp|P0479	MTERRVPI	57.56	IDP	0.6398	Yes	0.0151	No			2	Driver	3	-13.1	No	No	-0.57	-0.002
CDC73	Q6P1J9	>sp|Q6P1	MADVLSV	53.67	IDP	0.4019	No	0.4929	No		+	4	Client	14	-12.5	No	No	-0.58	0.046
SUMO2	P61956	>sp|P6195	MADEKPK	52.63	IDP	0.1814	No	0.4944	No		+	1	Client	2	-30.2	No	No	-0.89	-0.021
ML12A	P19105	>sp|P1910	MSSKRTKT	41.52	IDP	0.6107	Yes	0.0338	No			1	Driver	5	-25	No	No	-0.83	-0.064
XPO2	P55060	>sp|P5506	MELSDAN	12.05	Partial IDP	0.1253	No	0.1683	No		+	0	No	39	6.8	Yes	No	-0.03	-0.013
NOM1	Q5C9Z4	>sp|Q5C9	MAASRSA	55.47	IDP	0.9475	Yes	0.8288	Yes	+		5	Driver	19	-21.1	No	No	-0.64	0.016
BYST	Q13895	>sp|Q138	MPKFKAA	48.97	IDP	0.2822	No	0.1084	No		+	2	Client	15	-8.2	No	No	-0.37	0.015
SSF1	Q9NQ55	>sp|Q9NQ	MGQSGRS	54.55	IDP	0.7386	Yes	0.0358	No			4	Driver	12	-24.2	No	No	-0.76	0.079
NUMA1	Q14980	>sp|Q149	MTLHATR	94.42	IDP	1	Yes	0.6606	Yes	+		26	Driver	20	-34.9492	No	No	-0.92	-0.013
HS71B	P0DMV9	>sp|P0DM	MAKAAAI	29.64	Partial IDP	0.3928	No	0.1251	No		+	2	Client	18	-12.3	No	No	-0.38	-0.012
PPIA	P62937	>sp|P6293	MVNPTVF	20.61	Partial IDP	0.2089	No	0.0057	No		+	0	No	7	-6.7	No	No	-0.32	0.018
NOL10	Q9BSC4	>sp|Q9BS	MQVSSLN	44.62	IDP	0.2473	No	0.0748	No		+	2	Client	19	-15.6	No	No	-0.75	0.022
1433Z	P63104	>sp|P6310	MDKNELV	53.88	IDP	0.2169	No	0.0048	No		+	1	Client	6	-18.2	No	No	-0.62	-0.055
AATF	Q9NY61	>sp|Q9NY	MAGPQPL	65.71	IDP	0.7654	Yes	0.2619	No			4	Driver	8	-31.5	No	No	-0.91	-0.058
RRP1	P56182	>sp|P5618	MVSRVQL	50.76	IDP	0.7152	Yes	0.0183	No			2	Driver	10	-26.9	No	No	-0.84	0.029
PHF6	Q8IWS0	>sp|Q8IW	MSSSVEQ	54.52	IDP	0.8738	Yes	0.9423	Yes	+		4	Driver	6	-30.5	No	No	-1.01	0.067
NOC4L	Q9BVI4	>sp|Q9BV	MEREPGA	21.71	Partial IDP	0.3262	No	0.0045	No		+	2	Client	21	3.7	Yes	No	-0.08	0.018
ESF1	Q9H501	>sp|Q9H5	MSSKQEI	81.67	IDP	0.9412	Yes	0.43	No			10	Driver	11	-38.5	No	No	-1.23	-0.044
KRR1	Q13601	>sp|Q136	MASPSLER	55.64	IDP	0.6197	Yes	0.2739	No			4	Driver	10	-18.5	No	No	-0.78	0.084
HS90B	P08238	>sp|P0823	MPEEVHH	45.03	IDP	0.3869	No	0.2768	No		+	3	Client	18	-17.3	No	No	-0.68	-0.046
HNRPF	P52597	>sp|P5259	MMLGPEG	40.48	IDP	0.5243	No	0.0968	No		+	3	Client	10	-10.7	No	No	-0.47	-0.020
NOP14	P78316	>sp|P7831	MAKAKKV	70.36	IDP	0.859	Yes	0.8195	Yes	+		7	Driver	16	-23.3	No	No	-0.76	0.013
DX39A	O00148	>sp|O001	MAEQDVE	18.27	Partial IDP	0.1354	No	0.0079	No		+	1	Client	16	-4	No	No	-0.30	-0.016
HNRH1	P31943	>sp|P3194	MMLGTEG	51.22	IDP	0.9486	Yes	0.9727	Yes	+		3	Driver	9	-16	No	No	-0.60	-0.004
U2AF1	Q01081	>sp|Q010	MAEYLASI	45.83	IDP	0.3606	No	0.542	Yes			1	Client	6	-31.9	No	No	-1.06	0.048
K0020	Q15397	>sp|Q153	MEVKGKK	34.41	IDP	0.2025	No	0.3415	No		+	2	Client	23	-12	No	No	-0.56	0.076
BCCIP	Q9P287	>sp|Q9P2	MASRSKR	35.99	IDP	0.3828	No	0.6835	Yes			1	Client	8	-20.5	No	No	-0.69	-0.083
SERA	O43175	>sp|O431	MAFANLR	8.26	Ordered	0.1623	No	0.0149	No		+	1	Client	21	1.2	Yes	No	0.09	0.002
IF5A2	Q9GZV4	>sp|Q9GZ	MADEIDFT	35.95	IDP	0.2524	No	0.0061	No		+	1	Client	6	-9.4	No	No	-0.35	-0.020
ROA0	Q13151	>sp|Q131	MENSQLC	61.97	IDP	0.9818	Yes	0.9959	Yes	+		1	Driver	5	-20.5	No	No	-0.59	0.043
DDX49	Q9Y6V7	>sp|Q9Y6	MAGFAEL	21.95	Partial IDP	0.1582	No	0.0188	No		+	1	Client	19	-2.2	No	No	-0.18	0.041
TIF1B	Q13263	>sp|Q132	MAASAAA	63.11	IDP	0.9946	Yes	0.9829	Yes	+		6	Driver	18	-17.4	No	No	-0.39	-0.013
HNRH2	P55795	>sp|P5579	MMLSTEG	52.34	IDP	0.9768	Yes	0.9661	Yes	+		2	Driver	8	-15.4	No	No	-0.60	-0.004
NOP9	Q86U38	>sp|Q86U	MGQGPRS	32.86	IDP	0.4991	No	0.6737	Yes			3	Client	24	-4.7	No	No	-0.12	0.013
RL7	P18124	>sp|P1812	MEGVEEK	39.92	IDP	0.2086	No	0.4144	No		+	1	Client	8	-6.4	No	No	-0.62	0.169
NKRF	O15226	>sp|O152	MEKILQM	56.81	IDP	0.8482	Yes	0.4272	No			5	Driver	11	-18	No	No	-0.74	0.031
U3IP2	O43818	>sp|O438	MSATAAA	36.42	IDP	0.5052	No	0.2709	No		+	3	Client	13	-15.5	No	No	-0.42	0.020
GNL3	Q9BVP2	>sp|Q9BV	MKRPKLK	50.82	IDP	0.6868	Yes	0.5131	Yes	+		5	Driver	20	-18.9	No	No	-0.71	0.040
PCBP1	Q15365	>sp|Q153	MDAGVTE	36.52	IDP	0.3965	No	0.5417	Yes			2	Client	8	-4.9	No	No	-0.11	0.007
BOP1	Q14137	>sp|Q141	MAGSRGA	42.63	IDP	0.8092	Yes	0.2633	No			4	Driver	21	-13	No	No	-0.50	-0.007
RL13	P26373	>sp|P2637	MAPSRNG	81.04	IDP	0.7586	Yes	0.0102	No			2	Driver	3	-22	No	No	-0.85	0.194
TBB3	Q13509	>sp|Q135	MREIVHIQ	18.22	Partial IDP	0.2876	No	0.0108	No		+	2	Client	16	-7.6	No	No	-0.37	-0.043
DPM1	O60762	>sp|O607	MASLEVSF	15	Partial IDP	0.1368	No	0.0039	No		+	1	Client	10	1.6	Yes	No	-0.29	0.046
IF4A1	P60842	>sp|P6084	MSASQDS	11.58	Partial IDP	0.1437	No	0.0132	No		+	1	Client	15	-4.1	No	No	-0.26	-0.016
NOC2L	Q9Y3T9	>sp|Q9Y3	MAAAGSR	41.92	IDP	0.5919	No	0.4504	No		+	3	Client	20	-10.3	No	No	-0.43	-0.015
RRP1B	Q14684	>sp|Q146	MAPAMQ	73.09	IDP	0.9785	Yes	0.8648	Yes	+		7	Driver	8	-22.4	No	No	-0.76	0.061
MBB1A	Q9BQG0	>sp|Q9BQ	MESRDPA	40.36	IDP	0.835	Yes	0.4911	No			10	Driver	43	-11.4	No	No	-0.43	0.039
RBM39	Q14498	>sp|Q144	MADDIDIE	51.89	IDP	0.4127	No	0.5286	Yes			1	Client	17	-18.5	No	No	-0.65	0.069
NOC3L	Q8WTT2	>sp|Q8W	MKARRNK	53.25	IDP	0.713	Yes	0.1438	No			5	Driver	21	-10.1	No	No	-0.54	0.041
HEAT1	Q9H583	>sp|Q9H5	MTSLAQQ	15.53	Partial IDP	0.179	No	0.343	No		+	2	Client	95	9.517187	Yes	No	0.06	0.000
PESC	O00541	>sp|O005	MGGLEKK	49.49	IDP	0.6498	Yes	0.0241	No			4	Driver	16	-16.4	No	No	-0.69	0.009
RS8	P62241	>sp|P6224	MGISRDN	46.63	IDP	0.3264	No	0.0362	No		+	2	Client	4	-22.3	No	No	-1.02	0.173
HNRPR	O43390	>sp|O433	MANQVN	58.77	IDP	0.7561	Yes	0.9003	Yes	+		4	Driver	12	-24.4	No	No	-0.93	0.012
HNRH3	P31942	>sp|P3194	MDWVMK	63.29	IDP	0.9995	Yes	0.9924	Yes	+		3	Driver	6	-22	No	No	-0.73	0.006
SPB1	Q8IY81	>sp|Q8IY8	MGKKGKV	63.64	IDP	0.8852	Yes	0.5658	Yes	+		10	Driver	16	-25.8	No	No	-0.83	0.015
RBM14	Q96PK6	>sp|Q96P	MKIFVGN	81.32	IDP	0.9999	Yes	0.9903	Yes	+		6	Driver	7	-15.4	No	No	-0.45	0.043
RS3A	P61247	>sp|P6124	MAVGKNK	40.91	IDP	0.1592	No	0.806	Yes			2	Client	6	-15	No	No	-0.62	0.085
RL3	P39023	>sp|P3902	MSHRKFS	37.72	IDP	0.2835	No	0.0437	No		+	2	Client	11	-11.3	No	No	-0.65	0.136
TBB4B	P68371	>sp|P6837	MREIVHL	19.33	Partial IDP	0.2078	No	0.0074	No		+	1	Client	14	-7.4	No	No	-0.36	-0.045
TMA16	Q96EY4	>sp|Q96E	MPKAPKG	67.49	IDP	0.3322	No	0.0073	No		+	2	Client	1	-32.8	No	No	-1.09	0.057
HNRPM	P52272	>sp|P5227	MAAGVEA	76.3	IDP	0.9997	Yes	0.8675	Yes	+		8	Driver	18	-14	No	No	-0.34	0.015
CTBL1	Q8WYA6	>sp|Q8W	MDVGELL	42.98	IDP	0.2228	No	0.0399	No		+	2	Client	18	-16.6	No	No	-0.54	-0.049
GPTC4	Q5T3I0	>sp|Q5T3	MNVTPEV	86.32	IDP	0.9984	Yes	0.9327	Yes	+		3	Driver	2	-51.8	No	No	-1.49	0.070
TBB5	P07437	>sp|P0743	MREIVHIQ	18.92	Partial IDP	0.2479	No	0.0145	No		+	1	Client	15	-6.8	No	No	-0.35	-0.045
NOG1	Q9BZE4	>sp|Q9BZ	MAHYNFK	41.01	IDP	0.3117	No	0.0846	No		+	3	Client	14	-17.8	No	No	-0.75	0.062
NSA2	O95478	>sp|O954	MPQNEYIE	60	IDP	0.5957	No	0.1458	No		+	2	Client	7	-17.2	No	No	-0.87	0.165
PDS5A	Q29RF7	>sp|Q29R	MDFTAQP	33.21	IDP	0.4724	No	0.0229	No		+	4	Client	45	-7.5	No	No	-0.34	0.016
ECHA	P40939	>sp|P4093	MVACRAI	18.87	Partial IDP	0.1341	No	0.2273	No		+	1	Client	34	0.9	Yes	No	-0.08	0.032
ACTBL	Q562R1	>sp|Q562	MTDNELS	14.1	Partial IDP	0.1639	No	0.0223	No		+	0	No	16	0.8	Yes	No	-0.19	-0.016
RL32	P62910	>sp|P6291	MAALRPL	60.74	IDP	0.2298	No	0.0674	No		+	0	No	4	-14.5	No	No	-0.77	0.207
RL18	Q07020	>sp|Q070	MGVDIRH	48.94	IDP	0.5384	No	0.0087	No		+	2	Client	7	-14.8	No	No	-0.72	0.207
CEBPZ	Q03701	>sp|Q037	MAAVKEP	51.52	IDP	0.7525	Yes	0.3761	No			6	Driver	25	-19.1	No	No	-0.71	-0.011
RSSA	P08865	>sp|P0886	MSGALDV	33.56	IDP	0.6811	Yes	0.0101	No			2	Driver	11	-6.8	No	No	-0.31	-0.041
RL10L	Q96L21	>sp|Q96L	MGRRPAR	20.56	Partial IDP	0.1138	No	0.0048	No		+	0	No	6	-9.5	No	No	-0.52	0.129
NUCL	P19338	>sp|P1933	MVKLAKA	86.2	IDP	0.9872	Yes	0.941	Yes	+		7	Driver	14	-45.4	No	No	-1.13	-0.084
LPPRC	P42704	>sp|P4270	MAALLRS	15.06	Partial IDP	0.1614	No	0.0521	No		+	1	Client	44	-1.2	No	No	-0.21	-0.006
RL11	P62913	>sp|P6291	MAQDQG	16.29	Partial IDP	0.1553	No	0.0102	No		+	1	Client	5	-11.4	No	No	-0.53	0.070
DDX10	Q13206	>sp|Q132	MGKTANS	54.4	IDP	0.5356	No	0.2043	No		+	7	Client	20	-23.1	No	No	-0.83	0.019
PDS5B	Q9NTI5	>sp|Q9NT	MAHSKTR	39.6	IDP	0.7071	Yes	0.3699	No			4	Driver	41	-13.5	No	No	-0.57	0.025
HSP7C	P11142	>sp|P1114	MSKGPAV	38.7	IDP	0.3211	No	0.5824	Yes			2	Client	17	-13.8	No	No	-0.46	-0.015
URB2	Q14146	>sp|Q141	MAAVYSG	11.48	Partial IDP	0.1538	No	0.2375	No		+	1	Client	63	11.9	Yes	No	0.16	0.011
ESYT1	Q9BSJ8	>sp|Q9BS	MERSPGE	30.8	IDP	0.6037	Yes	0.1804	No			4	Driver	42	-4.6	No	No	-0.27	-0.011
LAP2A	P42166	>sp|P4216	MPEFLEDP	66.57	IDP	0.9877	Yes	0.9912	Yes	+		6	Driver	17	-17	No	No	-0.55	0.011

MGN2	Q96A72	>sp|Q96A	MAVASDF	16.22	Partial IDP	0.1468	No	0.0254	No		+	0	No	5	-1.5	No	No	-0.38	-0.003
NONO	Q15233	>sp|Q152	MQSNKTF	76.86	IDP	0.9869	Yes	0.609	Yes	+		4	Driver	8	-30.6	No	No	-1.00	0.025
RS26	P62854	>sp|P6285	MTKKRRN	50.43	IDP	0.8969	Yes	0.0222	No			2	Driver	3	-16.5	No	No	-0.66	0.204
NPM	P06748	>sp|P0674	MEDSMDN	61.22	IDP	0.9553	Yes	0.9884	Yes	+		2	Driver	8	-35.6	No	Yes	-0.97	-0.073
RRP8	O43159	>sp|O431	MFEEPEW	62.06	IDP	0.9406	Yes	0.9612	Yes	+		4	Driver	7	-24.3	No	No	-0.76	0.058
RBM8A	Q9Y5S9	>sp|Q9Y5	MADVLDL	61.49	IDP	0.9709	Yes	0.0354	No			3	Driver	3	-38	No	No	-1.15	-0.020
MCM3	P25205	>sp|P252	MAGTVVL	46.66	IDP	0.6128	Yes	0.2455	No			6	Driver	23	-16.8	No	No	-0.56	-0.014
RBM28	Q9NW13	>sp|Q9NV	MAGLTLFV	62.19	IDP	0.9274	Yes	0.4076	No			6	Driver	12	-30.8	No	No	-0.96	0.041
MATR3	P43243	>sp|P4324	MSKSFQQ	72.02	IDP	0.9982	Yes	0.9685	Yes	+		7	Driver	18	-29.2	No	No	-0.93	-0.006
RL4	P36578	>sp|P3657	MACARPLI	50.35	IDP	0.3958	No	0.0503	No		+	4	Client	11	-14.3	No	No	-0.61	0.177
RS9	P46781	>sp|P4678	MPVARSW	28.87	Partial IDP	0.2294	No	0.0171	No		+	1	Client	7	-13.4	No	No	-0.64	0.124
MCM4	P33991	>sp|P3399	MSSPASTP	35.46	IDP	0.324	No	0.6159	Yes			3	Client	29	-10.7	No	No	-0.41	0.003
DDX24	Q9GZR7	>sp|Q9GZ	MKLKDTKS	49.36	IDP	0.8808	Yes	0.8971	Yes	+		9	Driver	20	-19.1	No	No	-0.66	0.034
COPB	P53618	>sp|P5361	MTAAENV	23.82	Partial IDP	0.282	No	0.156	No		+	2	Client	34	1	Yes	No	-0.09	-0.008
HNRPC	P07910	>sp|P0791	MASNVTN	75.82	IDP	0.9961	Yes	0.7935	Yes	+		3	Driver	5	-35.4	No	No	-0.98	-0.036
SIN3A	Q96ST3	>sp|Q96ST	MKRRLDD	48.55	IDP	0.851	Yes	0.9604	Yes	+		8	Driver	29	-16.2	No	No	-0.69	0.012
RS24	P62847	>sp|P6284	MNDTVTIF	47.37	IDP	0.202	No	0.2207	No		+	1	Client	3	-21	No	No	-0.99	0.203
DDX50	Q9BQ39	>sp|Q9BQ	MPGKLLW	38.81	IDP	0.5348	No	0.9234	Yes			2	Client	22	-20.2	No	No	-0.71	0.037
ETFA	P13804	>sp|P1380	MFRAAAP	19.52	Partial IDP	0.2221	No	0.0106	No		+	1	Client	14	2.9	Yes	No	0.15	0.020
RL10A	P62906	>sp|P6290	MSSKVSRD	32.26	IDP	0.1548	No	0.0108	No		+	1	Client	6	-5.5	No	No	-0.41	0.111
MRT4	Q9UKD2	>sp|Q9UK	MPKSKRDI	32.22	IDP	0.2086	No	0.0172	No		+	1	Client	6	-15.8	No	No	-0.71	0.015
RL15	P61313	>sp|P6131	MGAYKYIC	51.47	IDP	0.6722	Yes	0.0107	No			2	Driver	5	-16.8	No	No	-0.93	0.218
SF3B3	Q15393	>sp|Q153	MFLYNLTL	16.68	Partial IDP	0.1376	No	0.0812	No		+	5	Client	47	-0.6	No	No	-0.17	-0.025
TBA1B	P68363	>sp|P6836	MRECISIH	16.63	Partial IDP	0.1976	No	0.0132	No		+	1	Client	19	-3.1	No	No	-0.23	-0.039
NOL11	Q9H8H0	>sp|Q9H8	MAALEEEF	21.97	Partial IDP	0.1517	No	0.1976	No		+	1	Client	29	4.1	Yes	No	-0.11	-0.008
RBM19	Q9Y4C8	>sp|Q9Y4	MSRLIVKN	58.96	IDP	0.9431	Yes	0.898	Yes	+		5	Driver	26	-22.7	No	No	-0.75	-0.001
RL17	P18621	>sp|P1862	MVRYSLDI	45.11	IDP	0.1848	No	0.0159	No		+	0	No	3	-21.2	No	No	-0.90	0.141
DDX54	Q8TDD1	>sp|Q8TD	MAADKGP	57.32	IDP	0.9391	Yes	0.6687	Yes	+		8	Driver	22	-21.1	No	No	-0.68	0.065
RL12	P30050	>sp|P3005	MPPKFDPI	23.64	Partial IDP	0.1717	No	0.0066	No		+	0	No	5	-12.6	No	No	-0.35	0.055
RPF2	Q9H7B2	>sp|Q9H7	MDTLDRV	47.39	IDP	0.2631	No	0.0298	No		+	1	Client	7	-11	No	No	-0.68	0.119
WDR3	Q9UNX4	>sp|Q9UN	MGLTKQY	28.53	Partial IDP	0.2985	No	0.0466	No		+	4	Client	36	-3.2	No	No	-0.26	0.002
OZF	Q15072	>sp|Q150	MSHLSQQ	69.52	IDP	0.269	No	0.1973	No		+	2	Client	4	-22.1	No	No	-0.88	0.106
DDX56	Q9NY93	>sp|Q9NY	MEDSEALC	30.71	IDP	0.2624	No	0.0198	No		+	2	Client	12	-4.8	No	No	-0.27	0.043
HSP76	P17066	>sp|P1706	MQAPREL	32.66	IDP	0.2901	No	0.2186	No		+	2	Client	17	-15	No	No	-0.46	-0.006
RPAB3	P52434	>sp|P5243	MAGILFED	25.33	Partial IDP	0.1667	No	0.0027	No		+	1	Client	6	-5.8	No	No	-0.34	-0.073
IF4A3	P38919	>sp|P3891	MATTATM	18.73	Partial IDP	0.1758	No	0.017	No		+	1	Client	15	-2.9	No	No	-0.26	0.001
MYH14	Q7Z406	>sp|Q7Z4	MAAVTMS	69.27	IDP	0.9776	Yes	0.5611	Yes	+		18	Driver	28	-29.2	No	No	-0.81	-0.016
CMS1	Q9BQ75	>sp|Q9BQ	MADDLGD	42.65	IDP	0.2969	No	0.0918	No		+	1	Client	9	-10.7	No	No	-0.50	0.043
WDR75	Q8IWA0	>sp|Q8IW	MVEEENIR	22.77	Partial IDP	0.2123	No	0.6001	Yes			1	Client	28	0.3	Yes	No	-0.25	-0.011
HNRPK	P61978	>sp|P6197	METEQPEI	56.8	IDP	0.9031	Yes	0.2392	No			4	Driver	9	-19.7	No	No	-0.70	-0.013
RL9	P32969	>sp|P3296	MKTILSNC	7.81	Ordered	0.092	No	0.0241	No		+	0	No	8	-4.3	No	No	-0.35	0.076
MK67I	Q9BYG3	>sp|Q9BY	MATFSGP	69.62	IDP	0.587	No	0.8185	Yes			4	Client	5	-21.1	No	No	-0.95	0.082
SMD3	P62318	>sp|P6231	MSIGVPIK	41.27	IDP	0.2632	No	0.0185	No		+	1	Client	6	-11.4	No	No	-0.43	0.103
RL18A	Q02543	>sp|Q025	MKASGTLI	38.64	IDP	0.1807	No	0.0071	No		+	1	Client	7	-7.4	No	No	-0.67	0.182
RS4X	P62701	>sp|P6270	MARGPKK	13.31	Partial IDP	0.1382	No	0.0053	No		+	0	No	10	-3.3	No	No	-0.33	0.106
ELAV1	Q15717	>sp|Q157	MSNGYED	25.46	Partial IDP	0.1844	No	0.0132	No		+	0	No	14	-6	No	No	-0.36	0.034
RL6	Q02878	>sp|Q028	MAGEKVE	61.46	IDP	0.5433	No	0.1071	No		+	2	Client	10	-12.9	No	No	-0.73	0.194
RL7A	P62424	>sp|P6242	MPKGKKA	41.73	IDP	0.3147	No	0.566	Yes			3	Client	10	-12.1	No	No	-0.55	0.162
WDR12	Q9GZL7	>sp|Q9GZ	MAQLQTR	20.09	Partial IDP	0.2042	No	0.0197	No		+	1	Client	11	-6	No	No	-0.39	-0.014
AP2B1	P63010	>sp|P6301	MTDSKYFT	20.49	Partial IDP	0.1488	No	0.1223	No		+	1	Client	40	2.2	Yes	No	-0.09	-0.019
RL8	P62917	>sp|P6291	MGRVIRG	44.75	IDP	0.3036	No	0.5649	Yes			2	Client	9	-13.9	No	No	-0.53	0.163
RL14	P50914	>sp|P5091	MVFRRFVI	59.07	IDP	0.3178	No	0.0519	No		+	2	Client	3	-16.9	No	No	-0.51	0.188
T2EA	P29083	>sp|P2908	MADPDVL	57.18	IDP	0.7727	Yes	0.0218	No			4	Driver	8	-22.1	No	No	-0.64	-0.067
MAK16	Q9BXY0	>sp|Q9BX	MQSDDVI	53.33	IDP	0.8313	Yes	0.0099	No			3	Driver	5	-30.9	No	No	-1.08	-0.025
RS2	P15880	>sp|P1588	MADDAGA	28.67	Partial IDP	0.3865	No	0.9444	Yes			2	Client	11	-4.3	No	No	-0.23	0.096
NOP2	P46087	>sp|P4608	MGRKLDP	64.78	IDP	0.988	Yes	0.9598	Yes	+		3	Driver	15	-26.2	No	No	-0.81	0.031
DDX52	Q9Y2R4	>sp|Q9Y2	MDVHDLF	32.72	IDP	0.328	No	0.9494	Yes			3	Client	23	-8.8	No	No	-0.44	0.061
PAK6	Q9NQU5	>sp|Q9NQ	MFRKKKKI	55.36	IDP	0.8415	Yes	0.7771	Yes	+		2	Driver	16	-11.7	No	No	-0.48	0.043
RS5	P46782	>sp|P4678	MTEWETA	25	Partial IDP	0.2268	No	0.0026	No		+	0	No	4	-9.6	No	No	-0.38	0.064
RS16	P62249	>sp|P6224	MPSKGPL	25.34	Partial IDP	0.1274	No	0.0277	No		+	1	Client	8	-5.8	No	No	-0.44	0.130
RALY	Q9UKM9	>sp|Q9UK	MSLKLQAS	65.36	IDP	0.8212	Yes	0.9803	Yes	+		2	Driver	7	-23.5	No	No	-0.64	0.026
PRP6	O94906	>sp|O949	MNKKKKP	41.02	IDP	0.5365	No	0.1299	No		+	4	Client	23	-18.7	No	No	-0.64	0.018
UTP15	Q8TED0	>sp|Q8TE	MAGYKPV	17.37	Partial IDP	0.1323	No	0.0501	No		+	1	Client	18	-0.6	No	No	-0.29	0.041
DCA13	Q9NV06	>sp|Q9NV	MKVKMLS	26.74	Partial IDP	0.156	No	0.0331	No		+	0	No	11	-8.3	No	No	-0.51	0.057
RS6	P62753	>sp|P6275	MKLNISFP	60.64	IDP	0.6884	Yes	0.3517	No			2	Driver	5	-24.4	No	No	-0.95	0.175
RS25	P62851	>sp|P6285	MPPKDDK	46.4	IDP	0.8546	Yes	0.0689	No			2	Driver	5	-26.9	No	No	-0.86	0.156
RL30	P62888	>sp|P6288	MVAAKKT	25.22	Partial IDP	0.1203	No	0.0147	No		+	1	Client	5	-2.3	No	No	-0.29	0.096
RS15A	P62244	>sp|P6224	MVRMNVI	16.15	Partial IDP	0.1088	No	0.004	No		+	0	No	8	2.2	Yes	No	-0.12	0.092
CENPV	Q7Z7K6	>sp|Q7Z7	MRRSRSSA	56.73	IDP	0.9263	Yes	0.9385	Yes	+		1	Driver	8	-23.3	No	No	-0.74	0.078

Table S2. Characteristics of the proteome of the acidosis-induced A-bodies. DPR are droplet-promoting regions, nHS are number of aggregation hot spots, Na4vSS are Normalized a4v Sequence Sum for 100 residues used for aggregation prediction.

Gene Name	UniProtID	Fasta	Sequence	PER(VSL2B)	Disorder Classifications	FuzDrop Score	FuzDrop LLPS	PSPredictor Score	PSPredictor LLPS	Fuz&PSP LLPS	Fuz&PSP No LLPS	Number of DPRs	Role in MLO	nHS	Na4vSS	Agregation Predicted	Aggregation DB scanning	Hydrophobicity	Charge
VHL	P40337	>sp|P4033	MPRRAEN	55.87	IDP	0.6579	Yes	0.0102	No			2	Driver	6	-23.0	No	Yes	-0.78	-0.073
AKAP13	Q12802	>sp|Q128	MKLNPQQ	75.97	IDP	1.0000	Yes	0.7561	Yes	+		30	Driver	46	-22.7	No	No	-0.64	-0.030
HERC2	O95714	>sp|O957	MPSESFCL	28.57	Partial IDP	0.9976	Yes	0.3504	No			28	Driver	153	-5.0	No	No	-0.19	-0.005
NOSIP	Q9Y314	>sp|Q9Y3	MTRHGKN	54.49	IDP	0.8464	Yes	0.0313	No			2	Driver	6	-15.1	No	No	-0.52	0.040
HSPB1	P04792	>sp|P0479	MTERRVPF	57.56	IDP	0.6398	Yes	0.0151	No			2	Driver	3	-13.1	No	No	-0.57	-0.002
PPIA	P62937	>sp|P6293	MVNPTVFF	20.61	Partial IDP	0.2089	No	0.0057	No		+	0	No	7	-6.7	No	No	-0.32	0.018
HSPA1A	P08107	>sp|P0810	MAKAAAIG	29.64	Partial IDP	0.3928	No	0.1251	No		+	2	Client	18	-12.3	No	No	-0.38	-0.012
CIRBP	Q14011	>sp|Q140	MASDEGKL	77.91	IDP	0.9977	Yes	0.9806	Yes	+		1	Driver	4	-32.7	No	No	-1.06	0.032
LYZ	P61626	>sp|P6162	MKALIVLG	8.11	Ordered	0.1266	No	0.0241	No		+	0	No	5	-2.6	No	Yes	-0.19	0.064
PABPN1	Q86U42	>sp|Q86U	MAAAAAA	76.14	IDP	0.9558	Yes	0.9338	Yes	+		2	Driver	4	-30.7	No	No	-0.82	-0.036
HNRNPA1	P09651	>sp|P0965	MSKSESPK	70.16	IDP	0.9998	Yes	0.9977	Yes	+		1	Driver	7	-27.8	No	No	-0.89	0.030
ANP32B	Q92688	>sp|Q926	MDMKRRI	61.75	IDP	0.4995	No	0.5683	Yes			1	Client	3	-47.7	No	No	-1.16	-0.263
PTBP1	P26599	>sp|P2659	MDGIVPDI	43.27	IDP	0.5386	No	0.9415	Yes			4	Client	25	-2.7	No	No	-0.12	0.030
PTGES3	Q15185	>sp|Q151	MQPASAK	56.88	IDP	0.7660	Yes	0.1522	No			1	Driver	4	-35.0	No	No	-1.05	-0.116
EEF1B2	P24534	>sp|P2453	MGFGDLKS	40.89	IDP	0.4031	No	0.1355	No		+	2	Client	7	-17.1	No	No	-0.49	-0.076
YBX1	P67809	>sp|P6780	MSSEAETQ	82.41	IDP	0.9970	Yes	0.984	Yes	+		2	Driver	1	-47.4	No	No	-1.48	0.049
CCT4	P50991	>sp|P5099	MPENVAPF	22.82	Partial IDP	0.2193	No	0.1655	No		+	1	Client	23	-0.1	No	No	0.03	0.009
SYMPK	Q92797	>sp|Q927	MASGSGDS	35.56	IDP	0.9426	Yes	0.5602	Yes	+		7	Driver	49	-3.9	No	No	-0.20	-0.005
ATRX	P46100	>sp|P4610	MTAEPMSI	72.03	IDP	0.9989	Yes	0.9671	Yes	+		17	Driver	37	-32.6	No	No	-1.06	0.000
TAF15	Q92804	>sp|Q928	MSDSGSYG	92.57	IDP	1.0000	Yes	0.9952	Yes	+		4	Driver	3	-50.1	No	No	-1.52	0.009
BLVRB	P30043	>sp|P3004	MAVKKIAIF	16.99	Partial IDP	0.1735	No	0.0317	No		+	1	Client	9	-1.2	No	No	-0.06	0.022
HNRNPH1	P31943	>sp|P3194	MMLGTEG	51.22	IDP	0.9486	Yes	0.9727	Yes	+		3	Driver	9	-16.0	No	No	-0.60	-0.004
HNRNPC	P07910	>sp|P0791	MASNVTN	75.82	IDP	0.9961	Yes	0.7935	Yes	+		3	Driver	5	-35.4	No	No	-0.98	-0.036
WDR46	O15213	>sp|O152	METAPKPG	47.05	IDP	0.7993	Yes	0.7594	Yes	+		4	Driver	19	-16.9	No	No	-0.60	0.062
NCL	P19338	>sp|P1933	MVKLAKAG	86.2	IDP	0.9872	Yes	0.941	Yes	+		7	Driver	14	-45.4	No	No	-1.13	-0.084
RSBN1	Q5VWQ0	>sp|Q5VW	MFISGRRT	51.87	IDP	0.8958	Yes	0.9117	Yes	+		8	Driver	15	-18.7	No	No	-0.66	0.032
YWHAZ	P63104	>sp|P6310	MDKNELV	53.88	IDP	0.2169	No	0.0048	No		+	1	Client	6	-18.2	No	No	-0.62	-0.055
FOXA1	P55317	>sp|P5531	MLGTVKM	83.9	IDP	0.9998	Yes	0.9673	Yes	+		4	Driver	12	-14.2	No	No	-0.48	0.028
ACTBL2	Q562R1	>sp|Q562	MTDNELSA	14.1	Partial IDP	0.1639	No	0.0223	No		+	0	No	16	0.8	Yes	No	-0.19	-0.016
XRCC6	P12956	>sp|P1295	MSGWESY	43.19	IDP	0.3735	No	0.4906	No		+	3	Client	19	-9.8	No	No	-0.54	0.001
HNRNPD	Q14103	>sp|Q141	MSEEQFG	83.1	IDP	0.9960	Yes	0.9949	Yes	+		2	Driver	7	-27.1	No	No	-0.92	0.010
HNRNPA3	P51991	>sp|P5199	MEVKPPPG	70.11	IDP	0.9994	Yes	0.9966	Yes	+		2	Driver	6	-30.9	No	No	-0.99	0.030
RPL29	P47914	>sp|P4791	MAKSKNHT	100	IDP	0.8048	Yes	0.6051	Yes	+		2	Driver	1	-34.1	No	No	-1.09	0.267
ZC3H18	Q86VM9	>sp|Q86V	MDVAESPE	92.86	IDP	0.9998	Yes	0.9929	Yes	+		4	Driver	5	-54.1	No	No	-1.59	0.010
ISY1	Q9ULR0	>sp|Q9UL	MARNAEK	72.98	IDP	0.5749	No	0.2225	No		+	1	Client	2	-31.0	No	No	-1.00	-0.037
HNRNPK	P61978	>sp|P6197	METEQPEE	56.8	IDP	0.9031	Yes	0.2392	No			4	Driver	9	-19.7	No	No	-0.70	-0.013
SEC22B	O75396	>sp|O7539	MVLLTMIA	32.09	IDP	0.2068	No	0.0114	No		+	1	Client	6	1.8	Yes	No	-0.18	0.021
HNRNPA2	P22626	>sp|P2262	MEKTLETV	69.97	IDP	0.9808	Yes	0.9977	Yes	+		2	Driver	4	-26.5	No	No	-0.93	0.024
TTLL12	Q14166	>sp|Q141	MEAERGPE	15.22	Partial IDP	0.2019	No	0.2262	No		+	3	Client	20	-5.3	No	No	-0.39	-0.025
PCBP1	Q15365	>sp|Q153	MDAGVTES	36.52	IDP	0.3965	No	0.5417	Yes			2	Client	8	-4.9	No	No	-0.11	0.007
HNRNPA0	Q13151	>sp|Q131	MENSQLCK	61.97	IDP	0.9818	Yes	0.9959	Yes	+		1	Driver	5	-20.5	No	No	-0.59	0.043
RBM3	P98179	>sp|P9817	MSSEEGKL	65.61	IDP	0.9952	Yes	0.9542	Yes	+		1	Driver	3	-32.4	No	No	-1.09	0.025
HNRNPCL1	O60812	>sp|O608	MASNVTN	67.58	IDP	0.9744	Yes	0.75	Yes	+		3	Driver	4	-34.8	No	No	-0.96	-0.039
RBM39	Q14498	>sp|Q144	MADDIDIE	51.89	IDP	0.4127	No	0.5286	Yes			1	Client	17	-18.5	No	No	-0.65	0.069
TAF6	P49848	>sp|P4984	MAEEKKLK	49.78	IDP	0.9420	Yes	0.9754	Yes	+		3	Driver	24	-7.1	No	No	-0.30	0.021
LUC7L2	Q9Y383	>sp|Q9Y3	MSAQAQM	74.49	IDP	0.9512	Yes	0.0666	No			3	Driver	2	-50.2	No	No	-1.49	0.088
DMD	P11532	>sp|P1153	MLWWEEV	53.57	IDP	0.8268	Yes	0.8378	Yes	+		15	Driver	83	-18.7	No	No	-0.69	-0.012
TADA2B	Q86TJ2	>sp|Q86T	MAELGKKY	48.57	IDP	0.3565	No	0.0117	No		+	2	Client	9	-17.7	No	No	-0.73	0.018
VPS13C	Q709C8	>sp|Q709	MVLESVVA	25.74	Partial IDP	0.7815	Yes	0.5435	Yes	+		11	Driver	133	0.1	Yes	No	-0.21	0.004
ELAVL1	Q15717	>sp|Q157	MSNGYED	25.46	Partial IDP	0.1844	No	0.0132	No		+	0	No	14	-6.0	No	No	-0.36	0.034
ACTB	P60709	>sp|P6070	MDDDIAAL	16.27	Partial IDP	0.2880	No	0.0145	No		+	0	No	14	-0.4	No	No	-0.20	-0.020
TPI1	P60174	>sp|P6017	MAPSRKFF	10.44	Partial IDP	0.1235	No	0.0406	No		+	0	No	10	-3.2	No	Yes	-0.12	0.004
DDX39A	O00148	>sp|O001	MAEQDVE	18.27	Partial IDP	0.1354	No	0.0079	No		+	1	Client	16	-4.0	No	No	-0.30	-0.016
MAGED2	Q9UNF1	>sp|Q9UN	MSDTSESG	72.11	IDP	0.9989	Yes	0.9609	Yes	+		5	Driver	11	-20.2	No	No	-0.53	0.029
RPL19	P84098	>sp|P8409	MSMLRLQ	67.86	IDP	0.5770	No	0.0112	No		+	2	Client	4	-28.5	No	No	-1.11	0.240
RFC1	P35251	>sp|P3525	MDIRKFFG	64.02	IDP	0.9781	Yes	0.7704	Yes	+		9	Driver	26	-24.4	No	No	-0.87	0.047
RPLP2	P05387	>sp|P0538	MRYVASYL	62.61	IDP	0.8018	Yes	0.7132	Yes	+		1	Driver	6	-20.5	No	No	-0.24	-0.078
SRSF2	Q01130	>sp|Q011	MSYGRPPP	83.71	IDP	0.9964	Yes	0.9881	Yes	+		1	Driver	2	-45.8	No	No	-1.62	0.206
C8orf33	Q9H7E9	>sp|Q9H7	MAALGHL	82.97	IDP	0.9495	Yes	0.4041	No			3	Driver	4	-29.0	No	No	-0.74	0.076
HNRNPM	P52272	>sp|P5227	MAAGVEA	76.3	IDP	0.9997	Yes	0.8675	Yes	+		8	Driver	18	-14.0	No	No	-0.34	0.015
SNRPD1	P62314	>sp|P6231	MKLVRFLM	34.45	IDP	0.3072	No	0.0353	No		+	1	Client	5	-14.7	No	No	-0.58	0.147
DDX6	P26196	>sp|P2619	MSTARTEN	27.54	Partial IDP	0.3308	No	0.3039	No		+	2	Client	20	-4.4	No	No	-0.32	0.026
ACTC1	P68032	>sp|P6803	MCDDEETT	16.71	Partial IDP	0.3178	No	0.0135	No		+	1	Client	13	-1.5	No	No	-0.23	-0.023
NAP1L1	P55209	>sp|P5520	MADIDNKE	56.52	IDP	0.7162	Yes	0.273	No			4	Driver	7	-30.4	No	No	-0.95	-0.130
PCBP2	Q15366	>sp|Q153	MDTGVIEG	44.93	IDP	0.9028	Yes	0.2466	No			3	Driver	10	-3.4	No	No	-0.14	0.003
RPS7	P62081	>sp|P6208	MFSSSAKI	44.85	IDP	0.2431	No	0.0164	No		+	2	Client	7	-8.4	No	No	-0.46	0.088
DDX5	P17844	>sp|P1784	MSGYSSDR	41.04	IDP	0.5261	No	0.4192	No		+	3	Client	18	-16.0	No	No	-0.64	0.029
MGMT	P16455	>sp|P1645	MDKDCEM	35.75	IDP	0.4840	No	0.0359	No		+	1	Client	5	-2.5	No	No	-0.04	0.027
GTF2F1	P35269	>sp|P3526	MAALGPSS	82.01	IDP	0.9976	Yes	0.9457	Yes	+		2	Driver	6	-43.4	No	No	-1.32	0.005
EIF4H	Q15056	>sp|Q150	MADFDTY	72.18	IDP	0.9945	Yes	0.856	Yes	+		2	Driver	3	-33.8	No	No	-1.01	0.002
SARNP	P82979	>sp|P8297	MATETVEL	99.05	IDP	0.9812	Yes	0.3577	No			4	Driver	4	-32.2	No	No	-0.91	-0.002
SF3A3	Q12874	>sp|Q128	METILEQQ	52.3	IDP	0.4000	No	0.0487	No		+	4	Client	9	-23.8	No	No	-0.92	-0.030
TRIM28	Q13263	>sp|Q132	MAASAAA	63.11	IDP	0.9946	Yes	0.9829	Yes	+		6	Driver	18	-17.4	No	No	-0.39	-0.013
SUGP1	Q8IWZ8	>sp|Q8IW	MSLKMDN	86.51	IDP	0.9973	Yes	0.9666	Yes	+		9	Driver	8	-25.8	No	No	-0.89	0.009
EIF3K	Q9UBQ5	>sp|Q9UB	MAMFEQM	16.06	Partial IDP	0.1250	No	0.0154	No		+	1	Client	10	0.2	Yes	No	-0.17	-0.044
SP1	P08047	>sp|P0804	MSDQDHS	79.24	IDP	1.0000	Yes	0.9946	Yes	+		7	Driver	20	-18.8	No	No	-0.44	0.008
TCEB3	Q14241	>sp|Q142	MHGGRSC	82.46	IDP	0.9986	Yes	0.9781	Yes	+		5	Driver	15	-31.0	No	No	-1.07	0.068
NCAPD3	P42695	>sp|P4269	MVALRGLG	36.25	IDP	0.7338	Yes	0.2037	No			7	Driver	53	-0.7	No	No	-0.20	0.015
HMCN2	Q8NDA2	>sp|Q8ND	MPGAPLLR	27.28	Partial IDP	0.5582	No	0.6315	Yes			10	Client	149	-6.2	No	No	-0.19	-0.011
NFRKB	Q6P4R8	>sp|Q6P4	MDSLDHM	72.52	IDP	0.9999	Yes	0.8222	Yes	+		15	Driver	39	-9.3	No	No	-0.32	0.026
SBNO1	A3KN83	>sp|A3KN	MVEPGQD	45.66	IDP	0.8679	Yes	0.5995	Yes	+		5	Driver	42	-11.8	No	No	-0.47	0.009
RGL4	Q8IZJ4	>sp|Q8IZJ	MRKLLTNL	44.82	IDP	0.3134	No	0.0136	No		+	1	Client	16	-8.0	No	No	-0.35	0.018
TNRC18	O15417	>sp|O154	MDGRDFG	89.96	IDP	1.0000	Yes	0.9261	Yes	+		24	Driver	39	-26.1	No	No	-0.70	0.021
HDGFRP2	Q7Z4V5	>sp|Q7Z4	MPHAFKPG	84.5	IDP	0.9983	Yes	0.9782	Yes	+		2	Driver	6	-49.8	No	No	-1.39	0.005
COMMD3	Q9UBI1	>sp|Q9UB	MELSESVQ	29.23	Partial IDP	0.1798	No	0.0098	No		+	1	Client	6	-9.0	No	No	-0.37	-0.013
TTN	Q8WZ42	>sp|Q8WZ	MTTQAPTF	31.29	IDP	1.0000	Yes	0.3769	No			12	Driver	935	-8.4	No	Yes	-0.45	-0.004
PRPF31	Q8WWY3	>sp|Q8W	MSLADELL	55.51	IDP	0.6981	Yes	0.655	Yes	+		3	Driver	15	-14.2	No	No	-0.49	-0.010
EIF5	P55010	>sp|P5501	MSVNVNR	51.51	IDP	0.7603	Yes	0.8132	Yes	+		6	Driver	9	-23.2	No	No	-0.80	-0.022
NAP1L4	Q99733	>sp|Q997	MADHSFSD	57.33	IDP	0.7910	Yes	0.2019	No			6	Driver	10	-23.6	No	No	-0.78	-0.087
COPE	O14579	>sp|O145	MAPPAPG	22.08	Partial IDP	0.2371	No	0.0444	No		+	1	Client	11	-11.0	No	No	-0.34	-0.034
DCD	P81605	>sp|P8160	MRFMTLLF	80.91	IDP	0.8322	Yes	0.1749	No			1	Driver	1	-18.9	No	No	-0.22	0.000
NQO1	P15559	>sp|P1555	MVGRRALI	19.34	Partial IDP	0.1890	No	0.0895	No		+	1	Client	13	3.2	Yes	No	-0.19	0.027
MBD3	O95983	>sp|O959	MERKRWE	68.73	IDP	0.7014	Yes	0.2176	No			2	Driver	6	-25.8	No	No	-0.79	-0.026
DHX9	Q08211	>sp|Q082	MGDVKNF	32.28	IDP	0.7863	Yes	0.1976	No			5	Driver	39	-5.4	No	No	-0.34	0.004
CD2BP2	O95400	>sp|O954	MPKRKVTF	71.55	IDP	0.9442	Yes	0.0895	No			6	Driver	2	-29.1	No	No	-0.83	-0.078

HBA1	P69905	>sp|P6990	MVLSPADK	20.42	Partial IDP	0.1494	No	0.0034	No		+	0	No	5	1.1	Yes	No	0.05	0.049
SNRPD3	P62318	>sp|P6231	MSIGVPIKV	41.27	IDP	0.2632	No	0.0185	No		+	1	Client	6	-11.4	No	No	-0.43	0.103
ZGPAT	Q8N5A5	>sp|Q8N5	MDEESLES	70.62	IDP	0.9725	Yes	0.746	Yes	+		6	Driver	12	-24.0	No	No	-0.59	-0.022
GATAD2B	Q8WXI9	>sp|Q8W	MDRMTED	83.14	IDP	0.9217	Yes	0.8894	Yes	+		6	Driver	11	-19.7	No	No	-0.61	0.046
UBE2E1	P51965	>sp|P5196	MSDDDSR	45.08	IDP	0.7531	Yes	0.1172	No			1	Driver	6	-12.5	No	No	-0.58	0.026
NOP9	Q86U38	>sp|Q86U	MGQGPRS	32.86	IDP	0.4991	No	0.6737	Yes			3	Client	24	-4.7	No	No	-0.12	0.013
CDK1	P06493	>sp|P0649	MEDYTKIEK	12.12	Partial IDP	0.1467	No	0.0056	No		+	0	No	11	2.2	Yes	No	-0.28	0.020
CHTOP	Q9Y3Y2	>sp|Q9Y3	MAAQSAP	100	IDP	0.9270	Yes	0.9882	Yes	+		3	Driver	2	-27.8	No	No	-0.71	0.159
SMNDC1	O75940	>sp|O759	MSEDLAKQ	76.89	IDP	0.8486	Yes	0.1218	No			3	Driver	3	-28.6	No	No	-0.92	0.004
SNRPGP15	A8MWD9	>sp|A8MV	MSKAHPPE	34.21	IDP	0.1100	No	0.0127	No		+	0	No	4	-5.5	No	No	-0.22	0.039
SNRPG	P62308	>sp|P6230	MSKAHPPE	32.89	IDP	0.1086	No	0.0068	No		+	0	No	3	-1.1	No	No	-0.07	0.039
RBM8A	Q9Y5S9	>sp|Q9Y5	MADVLDLH	61.49	IDP	0.9709	Yes	0.0354	No			3	Driver	3	-38.0	No	No	-1.15	-0.020
GSTM3	P21266	>sp|P2126	MSCESSMV	14.22	Partial IDP	0.1175	No	0.0047	No		+	0	No	9	-2.8	No	No	-0.41	-0.016
RPS17	P08708	>sp|P0870	MGRVRTKT	31.85	IDP	0.1601	No	0.009	No		+	1	Client	5	-11.4	No	No	-0.58	0.074
RPS17L	P0CW22	>sp|P0870	MGRVRTKT	31.85	IDP	0.1601	No	0.009	No		+	1	Client	5	-11.4	No	No	-0.58	0.074
HNRNPUL	Q9BUJ2	>sp|Q9BU	MDVRRLKV	63.79	IDP	0.9979	Yes	0.9796	Yes	+		4	Driver	14	-29.5	No	No	-1.08	0.003
RPS25	P62851	>sp|P6285	MPPKDDKK	46.4	IDP	0.8546	Yes	0.0689	No			2	Driver	5	-26.9	No	No	-0.86	0.156
C1QBP	Q07021	>sp|Q070	MLPLLRCV	62.41	IDP	0.3114	No	0.332	No		+	1	Client	12	-14.6	No	No	-0.46	-0.055
EWSR1	Q01844	>sp|Q018	MASTDYST	93.75	IDP	0.9999	Yes	0.9991	Yes	+		5	Driver	2	-34.4	No	No	-1.12	0.027
SNRPD2	P62316	>sp|P6231	MSLLNKPK	54.24	IDP	0.5876	No	0.177	No		+	2	Client	4	-15.7	No	No	-0.71	0.097
IGLC1	P0CG04	>sp|P0CG	GQPKANPT	71.7	IDP	0.2484	No	0.0161	No		+	0	No	4	-12.1	No	No	-0.52	0.019
IGLC2	P0CG05	>sp|P0CG	GQPKAAPS	74.53	IDP	0.3838	No	0.0419	No		+	1	Client	4	-9.8	No	No	-0.44	0.009
IGLC3	P0CG06	>sp|P0CG	GQPKAAPS	76.42	IDP	0.5929	No	0.0774	No		+	1	Client	4	-11.0	No	No	-0.46	0.009
IGLC6	P0CF74	>sp|P0CF7	GQPKAAPS	73.58	IDP	0.2728	No	0.0324	No		+	0	No	4	-11.1	No	No	-0.47	0.009
IGLC7	A0M8Q6	>sp|A0M8	GQPKAAPS	74.53	IDP	0.3412	No	0.0185	No		+	0	No	5	-11.6	No	No	-0.46	0.028
IGLL5	B9A064	>sp|B9A0	MRPKTGQ	69.16	IDP	0.7742	Yes	0.1969	No			2	Driver	7	-7.7	No	No	-0.40	0.035
POLA1	P09884	>sp|P0988	MAPVHGD	37.69	IDP	0.6683	Yes	0.1415	No			7	Driver	42	-8.7	No	No	-0.46	-0.011
RBBP4	Q09028	>sp|Q090	MADKEAAF	27.29	Partial IDP	0.3177	No	0.1343	No		+	3	Client	16	-15.6	No	No	-0.56	-0.064
IGKC	P01834	>sp|P0183	RTVAAPSVI	61.68	IDP	0.2708	No	0.0898	No		+	1	Client	4	-12.4	No	No	-0.54	0.000
ZFR	Q96KR1	>sp|Q96K	MIPICPVVS	74.49	IDP	0.9935	Yes	0.9789	Yes	+		11	Driver	24	-16.2	No	No	-0.56	0.037
HSPA8	P11142	>sp|P1114	MSKGPAVC	38.7	IDP	0.3211	No	0.5824	Yes			2	Client	17	-13.8	No	No	-0.46	-0.015
SRSF1	Q07955	>sp|Q079	MSGGGVIR	63.71	IDP	0.9496	Yes	0.9711	Yes	+		3	Driver	5	-31.8	No	No	-1.15	0.087
RPLP1	P05386	>sp|P0538	MASVSELA	50	IDP	0.9423	Yes	0.1345	No			1	Driver	5	-8.0	No	No	0.11	-0.092
DUSP3	P51452	>sp|P5145	MSGSFELS	17.84	Partial IDP	0.1135	No	0.0024	No		+	1	Client	6	-7.8	No	No	-0.31	0.014
EEF1D	P29692	>sp|P2969	MATNFLAH	62.99	IDP	0.9193	Yes	0.4908	No			3	Driver	6	-22.9	No	No	-0.59	-0.044
RPL31	P62899	>sp|P6289	MAPAKKG	30.4	IDP	0.2933	No	0.0207	No		+	1	Client	2	-16.7	No	No	-0.81	0.144
HMX3	A6NHT5	>sp|A6NH	MPEPGPDA	69.75	IDP	0.9069	Yes	0.6825	Yes	+		2	Driver	8	-18.8	No	No	-0.49	0.017
RPL11	P62913	>sp|P6291	MAQDQGE	16.29	Partial IDP	0.1553	No	0.0102	No		+	1	Client	5	-11.4	No	No	-0.53	0.070
SF3A1	Q15459	>sp|Q154	MPAGPVQ	77.68	IDP	0.9627	Yes	0.9809	Yes	+		6	Driver	14	-18.8	No	No	-0.74	-0.026
SNRNP40	Q96DI7	>sp|Q96D	MIEQQKRK	24.65	Partial IDP	0.2387	No	0.1868	No		+	2	Client	9	-8.1	No	No	-0.35	0.022
ESRP2	Q9H6T0	>sp|Q9H6	MTPPPPPP	32.6	IDP	0.3368	No	0.849	Yes			2	Client	25	-0.9	No	No	-0.09	0.002
RPS3	P23396	>sp|P2339	MAVQISKK	25.1	Partial IDP	0.2066	No	0.0055	No		+	1	Client	8	-0.1	No	No	-0.15	0.056
TRIM33	Q9UPN9	>sp|Q9UP	MAENKGG	65.04	IDP	0.9995	Yes	0.8915	Yes	+		9	Driver	21	-18.3	No	No	-0.54	0.003
FIP1L1	Q6UN15	>sp|Q6UN	MSAGEVER	77.1	IDP	0.9978	Yes	0.9323	Yes	+		4	Driver	6	-39.4	No	No	-1.25	-0.021
SRPRB	Q9Y5M8	>sp|Q9Y5	MASADSRF	29.52	Partial IDP	0.3636	No	0.0331	No		+	2	Client	9	-0.1	No	No	-0.02	0.024
CTDP1	Q9Y5B0	>sp|Q9Y5	MEVPAAGF	67.12	IDP	0.9981	Yes	0.972	Yes	+		5	Driver	16	-28.0	No	No	-0.74	-0.030
THOC6	Q86W42	>sp|Q86W	MERAVPLA	17.3	Partial IDP	0.1452	No	0.0652	No		+	0	No	12	-1.6	No	No	-0.16	0.015
DEK	P35659	>sp|P3565	MSASAPAA	80.53	IDP	0.9889	Yes	0.9115	Yes	+		2	Driver	4	-39.4	No	No	-1.29	0.020
COL5A1	P20908	>sp|P2090	MDVHTRW	79.71	IDP	0.9984	Yes	0.7971	Yes	+		3	Driver	18	-32.9	No	No	-0.87	-0.026
HAT1	O14929	>sp|O149	MAGFGAM	18.38	Partial IDP	0.1434	No	0.0051	No		+	0	No	11	-1.7	No	No	-0.45	-0.014
PPM1G	O15355	>sp|O153	MGAYLSQF	59.16	IDP	0.9759	Yes	0.3021	No			4	Driver	9	-33.1	No	No	-0.81	-0.130
SRSF5	Q13243	>sp|Q132	MSGCRVFI	69.49	IDP	0.9488	Yes	0.648	Yes	+		2	Driver	3	-38.3	No	No	-1.31	0.153
YWHAB	P31946	>sp|P3194	MTMDKSEI	54.47	IDP	0.2884	No	0.0169	No		+	1	Client	7	-20.8	No	No	-0.73	-0.053
CANX	P27824	>sp|P2782	MEGKWLL	54.39	IDP	0.7564	Yes	0.2416	No			3	Driver	14	-25.6	No	No	-0.87	-0.097
YBX3	P16989	>sp|P1698	MSEAGEAT	86.29	IDP	0.9974	Yes	0.9768	Yes	+		3	Driver	2	-38.1	No	No	-1.11	0.039
OTUB1	Q96FW1	>sp|Q96F	MAAEEPQ	25.83	Partial IDP	0.1198	No	0.0581	No		+	2	Client	8	-11.6	No	No	-0.55	-0.052
EEF1A1	P68104	>sp|P6810	MGKEKTHI	30.95	IDP	0.2234	No	0.0677	No		+	3	Client	19	-4.3	No	No	-0.26	0.035
EEF1A1P5	Q5VTE0	>sp|Q5VT	MGKEKTHI	32.25	IDP	0.2230	No	0.1452	No		+	3	Client	19	-4.0	No	No	-0.25	0.037
HNRNPL	P14866	>sp|P1486	MSRRLLPR	51.95	IDP	0.9229	Yes	0.9718	Yes	+		3	Driver	16	-17.3	No	No	-0.65	0.024
CALR	P27797	>sp|P2779	MLLSVPLLL	52.04	IDP	0.8045	Yes	0.2613	No			2	Driver	10	-33.7	No	No	-1.10	-0.133
RCOR1	Q9UKL0	>sp|Q9UK	MPAMVEK	58.97	IDP	0.9797	Yes	0.9427	Yes	+		3	Driver	6	-26.4	No	No	-0.76	0.005
RPL4	P36578	>sp|P3657	MACARPLI	50.35	IDP	0.3958	No	0.0503	No		+	4	Client	11	-14.3	No	No	-0.61	0.177
NIFK	Q9BYG3	>sp|Q9BY	MATFSGPA	69.62	IDP	0.5870	No	0.8185	Yes			4	Client	5	-21.1	No	No	-0.95	0.082
MDH2	P40926	>sp|P4092	MLSALARP	22.78	Partial IDP	0.2995	No	0.0113	No		+	0	No	14	3.9	Yes	No	0.14	0.028
RPL23A	P62750	>sp|P6275	MAPKAKKE	48.72	IDP	0.6353	Yes	0.0151	No			2	Driver	5	-18.5	No	No	-0.73	0.186
SUGP2	Q8IX01	>sp|Q8IX0	MAARRITQ	62.75	IDP	0.9879	Yes	0.6401	Yes	+		11	Driver	18	-19.1	No	No	-0.64	0.008
POLD3	Q15054	>sp|Q150	MADQLYLE	74.89	IDP	0.9966	Yes	0.9953	Yes	+		4	Driver	7	-24.2	No	No	-0.85	0.048
CSTF2	P33240	>sp|P3324	MAGLTVRD	81.11	IDP	0.9942	Yes	0.9489	Yes	+		1	Driver	10	-20.2	No	No	-0.50	0.003
INO80E	Q8NBZ0	>sp|Q8NB	MNGPADG	93.85	IDP	0.9953	Yes	0.4419	No			2	Driver	8	-20.6	No	No	-0.71	0.010
RRP12	Q5JTH9	>sp|Q5JTH	MGRSGKLF	36.24	IDP	0.5879	No	0.5229	Yes			7	Client	44	-4.9	No	No	-0.23	0.032
G3BP1	Q13283	>sp|Q132	MVMEKPSI	63.95	IDP	0.9937	Yes	0.4903	No			2	Driver	10	-24.7	No	No	-0.83	-0.021
ARID1A	O14497	>sp|O144	MAAQVAP	80.18	IDP	1.0000	Yes	0.9758	Yes	+		8	Driver	28	-24.6	No	No	-0.78	0.002
KPNA4	O00629	>sp|O006	MADNEKLD	28.21	Partial IDP	0.1335	No	0.4221	No		+	0	No	22	-5.0	No	No	-0.16	-0.042
HSPA12B	Q96MM6	>sp|Q96M	MLAVPEM	24.05	Partial IDP	0.2691	No	0.2726	No		+	2	Client	23	-4.5	No	No	-0.22	0.024
RPL12	P30050	>sp|P3005	MPPKFDPN	23.64	Partial IDP	0.1717	No	0.0066	No		+	0	No	5	-12.6	No	No	-0.35	0.055
HNRNPR	O43390	>sp|O433	MANQVNG	58.77	IDP	0.7561	Yes	0.9003	Yes	+		4	Driver	12	-24.4	No	No	-0.93	0.012
RPL18A	Q02543	>sp|Q025	MKASGTLR	38.64	IDP	0.1807	No	0.0071	No		+	1	Client	7	-7.4	No	No	-0.67	0.182
ETFA	P13804	>sp|P1380	MFRAAAPC	19.52	Partial IDP	0.2221	No	0.0106	No		+	1	Client	14	2.9	Yes	No	0.15	0.020
LEMD2	Q8NC56	>sp|Q8NC	MAGLSDLE	48.31	IDP	0.6818	Yes	0.0125	No			2	Driver	9	-12.2	No	No	-0.47	0.023
TUBB3	Q13509	>sp|Q135	MREIVHIQ	18.22	Partial IDP	0.2876	No	0.0108	No		+	2	Client	16	-7.6	No	No	-0.37	-0.043
AKAP8	O43823	>sp|O438	MDQGYGG	86.42	IDP	0.9990	Yes	0.9912	Yes	+		7	Driver	7	-35.1	No	No	-1.00	-0.035
LACRT	Q9GZZ8	>sp|Q9GZ	MKFTTLLFL	73.19	IDP	0.7637	Yes	0.2452	No			1	Driver	3	-12.7	No	No	-0.25	-0.011
HDAC2	Q92769	>sp|Q927	MAYSQGG	36.68	IDP	0.2178	No	0.2059	No		+	1	Client	13	-17.5	No	No	-0.72	-0.014
RPL10A	P62906	>sp|P6290	MSSKVSRD	32.26	IDP	0.1548	No	0.0108	No		+	1	Client	6	-5.5	No	No	-0.41	0.111
FGB	P02675	>sp|P0267	MKRMVSW	37.07	IDP	0.3645	No	0.3233	No		+	2	Client	7	-16.9	No	No	-0.76	0.021
DDX39B	Q13838	>sp|Q138	MAENDVD	20.79	Partial IDP	0.1150	No	0.0115	Yes			2	Client	14	-4.4	No	No	-0.27	-0.018
PRPF4	O43172	>sp|O431	MASSRASS	37.16	IDP	0.3376	No	0.092	No		+	3	Client	13	-12.7	No	No	-0.49	0.017
POLDIP3	Q9BY77	>sp|Q9BY	MADISLDEI	76.48	IDP	0.3363	No	0.8247	No		+	2	Client	10	-15.1	No	No	-0.47	0.071
COMT	P21964	>sp|P2196	MPEAPPLL	7.01	Ordered	0.1043	No	0.0046	No		+	0	No	9	9.1	Yes	No	0.16	-0.022
NELFCD	Q8IXH7	>sp|Q8IXH	MAGAVPG	18.47	Partial IDP	0.2366	No	0.0383	No		+	1	Client	25	-0.2	No	No	-0.13	-0.039

Table S3. Characteristics of the proteome of the nSBs. DPR are droplet-promoting regions, nHS are number of aggregation hot spots, Na4vSS are Normalized a4v Sequence Sum for 100 residues used for aggregation prediction.

Gene Name	UniProtID	Fasta	Sequence	PER(VSL2B)	Disorder Classification	FuzDrop Score	FuzDrop LLPS	PSPredictor Score	PSPredictor LLPS	Fuz&PSP LLPS	Fuz&PSP No LLPS	Number of DPRs	Role in MLO	nHS	Na4vSS	Agregation Predicted	Aggregation on DB	Hydrophobicity	Charge
HSF1	Q00613	>sp|Q006	MDLPVGPG	72.97	IDP	0.9884	Yes	0.9457	Yes	+		5	Driver	15	-0.112	No	No	-0.39	-0.029
HSF2	Q03933	>sp|Q039	MKQSSNVP	67.35	IDP	0.8074	Yes	0.8322	Yes	+		5	Driver	11	-0.16	No	No	-0.58	-0.054
SLTM	Q9NWH9	>sp|Q9NV	MAAATGAV	92.65	IDP	0.9998	Yes	0.9665	Yes	+		6	Driver	6	-50	No	No	-1.45	0.015
NCOA5	Q9HCD5	>sp|Q9HC	MNTAPSRP	81.52	IDP	0.9973	Yes	0.7903	Yes	+		5	Driver	8	-35.4	No	No	-1.10	0.032
DHX9	Q08211	>sp|Q082	MGDVKNFL	32.28	IDP	0.7863	Yes	0.1976	No			5	Driver	39	-5.4	No	No	-0.34	0.004
ZNF638	Q14966	>sp|Q149	MSRPRFNP	81.24	IDP	0.9954	Yes	0.9458	Yes	+		20	Driver	34	-24.3	No	No	-0.81	-0.003
HNRNPM	P52272	>sp|P5227	MAAGVEAA	76.30	IDP	0.9997	Yes	0.8675	Yes	+		8	Driver	18	-14	No	No	-0.34	0.015
SRSF1	Q07955	>sp|Q079	MSGGGVIR	63.71	IDP	0.9496	Yes	0.9711	Yes	+		3	Driver	5	-31.8	No	No	-1.15	0.087
TRA2B	P62995	>sp|P6299	MSDSGEQN	79.17	IDP	0.9948	Yes	0.9957	Yes	+		3	Driver	4	-42.9	No	No	-1.62	0.158
SAFB2	Q14151	>sp|Q141	MAETLPGSG	93.18	IDP	0.9996	Yes	0.7464	Yes	+		7	Driver	6	-45.5	No	No	-1.29	-0.009
RBMX	P38159	>sp|P3815	MVEADRPG	86.96	IDP	0.9984	Yes	0.9984	Yes	+		1	Driver	3	-40.5	No	No	-1.37	0.063
RBMXL1	Q96E39	>sp|Q96E	MVEADRPG	86.92	IDP	0.9984	Yes	0.998	Yes	+		2	Driver	3	-38.2	No	No	-1.29	0.056
SRSF9	Q13242	>sp|Q132	MSGWADER	52.04	IDP	0.8195	Yes	0.0665	No			3	Driver	4	-25.3	No	No	-1.07	0.023
DDX39A	O00148	>sp|O001	MAEQDVEN	18.27	Partial IDP	0.1354	No	0.0079	No		+	1	Client	16	-4	No	No	-0.30	-0.016
DDX39B	Q13838	>sp|Q138	MAENDVDN	20.79	Partial IDP	0.115	No	0.0115	No		+	2	Client	14	-4.4	No	No	-0.27	-0.018
SRSF7	Q16629	>sp|Q166	MSRYGRYG	81.09	IDP	0.9857	Yes	0.9955	Yes	+		2	Driver	5	-36.7	No	No	-1.40	0.200
THRAP3	Q9Y2W1	>sp|Q9Y2	MSKTNKSKS	97.91	IDP	0.9996	Yes	0.998	Yes	+		2	Driver	4	-48.8	No	No	-1.63	0.097
POLDIP3	Q9BY77	>sp|Q9BY	MADISLDEL	76.48	IDP	0.3363	No	0.8247	Yes			2	Client	10	-15.1	No	No	-0.47	0.071
RBM15	Q96T37	>sp|Q96T	MRTAGRDP	68.78	IDP	0.9996	Yes	0.891	Yes	+		6	Driver	14	-27.8	No	No	-0.87	0.058
BCLAF1	Q9NYF8	>sp|Q9NY	MGRSNSRS	96.96	IDP	0.9995	Yes	0.9979	Yes	+		11	Driver	4	-47.3	No	No	-1.63	0.088
SRSF2	Q01130	>sp|Q011	MSYGRPPP	83.71	IDP	0.9964	Yes	0.9881	Yes	+		1	Driver	2	-45.8	No	No	-1.62	0.206
ALYREF	Q86V81	>sp|Q86V	MADKMDM	66.54	IDP	0.996	Yes	0.986	Yes	+		2	Driver	3	-35	No	No	-0.82	0.080
HNRNPA2	P22626	>sp|P2262	MEKTLETVP	69.97	IDP	0.9808	Yes	0.9977	Yes	+		2	Driver	4	-26.5	No	No	-0.93	0.024
SRSF3	P84103	>sp|P841	MHRDSCPL	74.39	IDP	0.9945	Yes	0.8645	Yes	+		1	Driver	4	-43.2	No	No	-1.52	0.165
RBMXL2	O75526	>sp|O755	MVEADRPG	86.73	IDP	0.9981	Yes	0.9385	Yes	+		1	Driver	3	-38.5	No	No	-1.25	0.080
RBMXL3	Q8N7X1	>sp|Q8N7	MMEADRPE	93.35	IDP	0.9997	Yes	0.996	Yes	+		3	Driver	7	-42.1	No	No	-1.37	0.047
LUC7L3	O95232	>sp|O952	MISAAQLLD	77.31	IDP	0.9957	Yes	0.6483	Yes	+		2	Driver	4	-55.1	No	No	-1.70	0.074
YTHDC1	Q96MU7	>sp|Q96M	MAADSREE	81.71	IDP	0.9987	Yes	0.9083	Yes	+		3	Driver	8	-47.8	No	No	-1.52	-0.008
SRSF5	Q13243	>sp|Q132	MSGCRVFIG	69.49	IDP	0.9488	Yes	0.648	Yes	+		2	Driver	3	-38.3	No	No	-1.31	0.153
RBM12B	Q8IXT5	>sp|Q8IXT	MAVVIRLLG	64.84	IDP	0.9936	Yes	0.7464	Yes	+		6	Driver	23	-28.8	No	No	-1.08	0.006
DDX5	P17844	>sp|P1784	MSGYSSDRD	41.04	IDP	0.5261	No	0.4192	No		+	3	Client	18	-16	No	No	-0.64	0.029
C7orf55-LU	Q96HJ9	>sp|Q96H	MAALGSPSH	35.40	IDP	0.6512	Yes	0.0023	No			1	Driver	3	-10.8	No	No	-0.47	0.115
SRSF4	Q08170	>sp|Q081	MPRVYIGRL	77.33	IDP	0.9972	Yes	0.9944	Yes	+		2	Driver	5	-55.3	No	No	-1.84	0.197
SRSF6	Q13247	>sp|Q132	MPRVYIGRL	70.35	IDP	0.9978	Yes	0.998	Yes	+		2	Driver	4	-43.4	No	No	-1.55	0.170
PPHLN1	Q8NEY8	>sp|Q8NE	MWSEGRYE	85.59	IDP	0.9978	Yes	0.91	Yes	+		4	Driver	4	-36.1	No	No	-1.30	0.032
CPSF7	Q8N684	>sp|Q8N6	MSEGVDLID	74.95	IDP	0.9987	Yes	0.9906	Yes	+		5	Driver	12	-22.6	No	No	-0.78	0.020
SPEN	Q96T58	>sp|Q96T	MVRETRHLV	84.12	IDP	1	Yes	0.8979	Yes	+		24	Driver	55	-27.3501	No	No	-0.86	0.011
ZC3H13	Q5T200	>sp|Q5T2	MSKIRRKVT	94.18	IDP	0.9997	Yes	0.8977	Yes	+		5	Driver	10	-61.8	No	No	-1.86	0.041
SRSF10	O75494	>sp|O754	MSRYLRPPN	73.28	IDP	0.9947	Yes	0.979	Yes	+		1	Driver	4	-45.3	No	No	-1.76	0.179
PPIA	P62937	>sp|P6293	MVNPTVFFD	20.61	Partial IDP	0.2089	No	0.0057	No		+	0	No	7	-6.7	No	No	-0.32	0.018
U2AF2	P26368	>sp|P2636	MSDFDEFER	42.74	IDP	0.3891	No	0.0708	No		+	2	Client	14	-15	No	No	-0.59	0.031
DDX17	Q92841	>sp|Q928	MPTGFVAPI	46.36	IDP	0.8739	Yes	0.856	Yes	+		3	Driver	21	-16.8	No	No	-0.61	0.016
HNRNPA1	P09651	>sp|P0965	MSKSESPKE	70.16	IDP	0.9998	Yes	0.9977	Yes	+		1	Driver	7	-27.8	No	No	-0.89	0.030
HNRNPA1	Q32P51	>sp|Q32P	MSKSASPKE	63.13	IDP	0.9711	Yes	0.9958	Yes	+		1	Driver	7	-25.6	No	No	-0.84	0.031
HNRNPK	P61978	>sp|P6197	METEQPEET	56.80	IDP	0.9031	Yes	0.2392	No			4	Driver	9	-19.7	No	No	-0.70	-0.013
SRSF12	Q8WXF0	>sp|Q8W	MSRYTRPPN	73.18	IDP	0.9941	Yes	0.9897	Yes	+		1	Driver	3	-40.1	No	No	-1.53	0.193
LUC7L	Q9NQ29	>sp|Q9NQ	MSAQAQMI	74.66	IDP	0.944	Yes	0.0191	No			2	Driver	3	-45.7	No	No	-1.34	0.075
LUC7L2	Q9Y383	>sp|Q9Y3	MSAQAQMI	74.79	IDP	0.9512	Yes	0.0666	No			2	Driver	2	-50.2	No	No	-0.49	0.017
FYTTD1	Q96QD9	>sp|Q96Q	KVHDLALRA	71.38	IDP	0.6429	Yes	0.4269	No			3	Driver	5	-21.8	No	No	-0.52	-0.100
TRA2A	Q13595	>sp|Q135	AKAERVHEL	80.50	IDP	0.9968	Yes	0.9839	Yes	+		2	Driver	4	-41.3	No	No	-0.97	-0.042
DNAJC14	Q6Y2X3	>sp|Q6Y2	SFQQQKLRV	55.41	IDP	0.9019	Yes	0.5774	Yes	+		4	Driver	11	-18.4	No	No	-0.76	0.058
SARNP	P82979	>sp|P8297	QERLKRREE	99.05	IDP	0.9812	Yes	0.3577	No			4	Driver	4	-32.2	No	No	-3.05	0.358
HNRNPA3	P51991	>sp|P5199	HRHRSRSSS	70.11	IDP	0.9994	Yes	0.9966	Yes	+		2	Driver	6	-30.9	No	No	-2.59	0.283
HNRNPF	P52597	>sp|P5259	DRDRKDKKR	40.48	IDP	0.5243	No	0.0968	No		+	3	Client	10	-10.7	No	No	-2.49	0.000
SRRM1	Q8IYB3	>sp|Q8IYB	MDAGFFRG	90.27	IDP	0.9985	Yes	0.9964	Yes	+		2	Driver	7	-48.3	No	No	-1.66	0.192
CHTOP	Q9Y3Y2	>sp|Q9Y3	MAAQSAPK	100.00	IDP	0.927	Yes	0.9882	Yes	+		3	Driver	2	-27.8	No	No	-0.71	0.159
SRSF8	Q9BRL6	>sp|Q9BR	MSCGRPPPD	84.75	IDP	0.9973	Yes	0.9973	Yes	+		1	Driver	3	-41.7	No	No	-1.58	0.199
CPSF6	Q16630	>sp|Q166	MADGVDHI	79.31	IDP	0.9976	Yes	0.9637	Yes	+		4	Driver	5	-30.4	No	No	-0.97	0.007
NUDT21	O43809	>sp|O438	MSVVPPNRS	19.82	Partial IDP	0.134	No	0.0115	No		+	1	Client	12	-0.5	No	No	-0.40	0.024
SFPQ	P23246	>sp|P2324	MSRDRFRSR	79.77	IDP	0.999	Yes	0.9964	Yes	+		3	Driver	7	-36.4	No	No	-1.13	0.031
RBM39	Q14498	>sp|Q144	MADDIDIEA	51.89	IDP	0.4127	No	0.5286	Yes			1	Client	17	-18.5	No	No	-0.65	0.069
EIF4A3	P38919	>sp|P3891	MATTATMA	18.73	Partial IDP	0.1758	No	0.017	No		+	1	Client	15	-2.9	No	No	-0.26	0.001
HNRNPH1	P31943	>sp|P3194	MMLGTEGG	51.22	IDP	0.9486	Yes	0.9727	Yes	+		3	Driver	9	-16	No	No	-0.60	-0.004
HNRNPH2	P55795	>sp|P5579	MMLSTEGR	52.34	IDP	0.9768	Yes	0.9661	Yes	+		2	Driver	8	-15.4	No	No	-0.60	-0.004
NONO	Q15233	>sp|Q152	MQSNKTFN	76.86	IDP	0.9869	Yes	0.609	Yes	+		4	Driver	8	-30.6	No	No	-1.00	0.025
CFL1	P23528	>sp|P2352	MASGVAVS	36.75	IDP	0.2136	No	0.0245	No		+	0	No	9	-6.9	No	No	-0.36	0.015
DDX23	Q9BUQ8	>sp|Q9BU	MAGELADK	54.27	IDP	0.7561	Yes	0.1926	No			2	Driver	17	-35.4	No	No	-1.15	0.051
TARDBP	Q13148	>sp|Q131	MSEYIRVTED	57.25	IDP	0.8981	Yes	0.9845	Yes	+		1	Driver	11	-17.1	No	Yes	-0.53	-0.004
WTAP	Q15007	>sp|Q150	MTNEEPLPK	86.11	IDP	0.9367	Yes	0.9979	Yes	+		2	Driver	5	-33.5	No	No	-1.00	-0.025
FUS	P35637	>sp|P3563	MASNDYTQ	90.68	IDP	0.9999	Yes	0.9987	Yes	+		3	Driver	2	-44.3	No	Yes	-1.32	0.029
TAF15	Q92804	>sp|Q928	MSDSGSYG	92.57	IDP	1	Yes	0.9952	Yes	+		4	Driver	3	-50.1	No	No	-1.52	0.009
ARGLU1	Q9NWB6	>sp|Q9NV	MGRSRSRSS	100.00	IDP	0.9913	Yes	0.2733	No			5	Driver	0	-58.4	No	No	-1.83	0.097
HNRNPU	Q00839	>sp|Q008	MSSSPVNVK	61.94	IDP	0.9928	Yes	0.9934	Yes	+		3	Driver	15	-32.4	No	No	-0.98	-0.008
SAP18	O00422	>sp|O004	MAVESRVTC	62.75	IDP	0.4504	No	0.0159	No		+	2	Client	5	-17.4	No	No	-0.77	0.042
API5	Q9BZZ5	>sp|Q9BZ	MPTVEELYR	32.82	IDP	0.2881	No	0.267	No		+	2	Client	16	-8.2	No	No	-0.39	0.005
HNRNPAB	Q99729	>sp|Q997	MSEAGEEQ	81.33	IDP	0.9939	Yes	0.991	Yes	+		2	Driver	8	-28	No	No	-1.01	0.012
HNRNPDL	O14979	>sp|O149	MEVPPRLSH	76.90	IDP	0.9972	Yes	0.9932	Yes	+		5	Driver	6	-26.4	No	Yes	-0.95	0.052
NCL	P19338	>sp|P1933	MVKLAKAG	86.20	IDP	0.9872	Yes	0.941	Yes	+		7	Driver	14	-45.4	No	No	-1.13	-0.084
HNRNPH3	P31942	>sp|P3194	MDWVMKH	63.29	IDP	0.9995	Yes	0.9924	Yes	+		3	Driver	6	-22	No	No	-0.73	0.006
KHSRP	Q92945	>sp|Q929	MSDYSTGG	78.06	IDP	0.9978	Yes	0.9936	Yes	+		9	Driver	10	-27.3	No	No	-0.77	0.006
S100A4	P26447	>sp|P2644	MACPLEKAL	57.43	IDP	0.1621	No	0.0054	No		+	0	No	2	-13.4	No	No	-0.56	-0.005
S100A6	P06703	>sp|P0670	MACPLDQA	48.89	IDP	0.1219	No	0.003	No		+	0	No	2	-10.2	No	No	-0.29	-0.022
SNRNP70	P08621	>sp|P0862	MTQFLPPNL	86.04	IDP	0.9968	Yes	0.5136	Yes	+		3	Driver	4	-60.4	No	No	-1.84	0.057
SRRT	Q9BXP5	>sp|Q9BX	MGDSDDEY	68.49	IDP	0.9913	Yes	0.8655	Yes	+		6	Driver	16	-33.9	No	No	-1.13	-0.011
TXN	P10599	>sp|P1059	MVKQIESKT	30.48	IDP	0.0927	No	0.0563	No		+	0	No	4	-1.7	No	No	-0.10	-0.043
DDX46	Q7L014	>sp|Q7L0	MGRESRHY	48.69	IDP	0.6304	Yes	0.4611	No			4	Driver	26	-28.1	No	No	-0.93	0.034
HNRNPL	P14866	>sp|P1486	MSRRLLPRA	51.95	IDP	0.9229	Yes	0.9718	Yes	+		3	Driver	16	-17.3	No	No	-0.65	0.024
PFN1	P07737	>sp|P0773	MAGWNAYI	15.00	Partial IDP	0.1044	No	0.018	No		+	0	No	6	-1	No	No	-0.12	0.021
RBM3	P98179	>sp|P9817	MSSEEGKLF	65.61	IDP	0.9952	Yes	0.9542	Yes	+		1	Driver	3	-32.4	No	No	-1.09	0.025
TBX18	O95935	>sp|O959	MAEKRRGS	66.39	IDP	0.9839	Yes	0.9943	Yes	+		5	Driver	16	-12.9	No	No	-0.44	0.028
ZC3H18	Q86VM9	>sp|Q86V	MDVAESPEF	92.86	IDP	0.9998	Yes	0.9929	Yes	+		4	Driver	5	-54.1	No	No	-1.59	0.010
CBX3	Q13185	>sp|Q131	MASNKTTLC	57.92	IDP	0.9497	Yes	0.034	No			3	Driver	4	-30.9	No	No	-0.97	-0.025
CIRBP	Q14011	>sp|Q140	MASDEGKLF	77.91	IDP	0.9977	Yes	0.9806	Yes	+		1	Driver	4	-32.7	No	No	-1.06	0.032
CTCFL	Q8NI51	>sp|Q8NI5	MAATEISVLS	68.63	IDP	0.6967	Yes	0.8801	Yes	+		4	Driver	17	-21.4	No	No	-0.77	0.044
HMGB1	P09429	>sp|P0942	MGKGDPKK	91.16	IDP	0.8945	Yes	0.8837	Yes	+		3	Driver	3	-51.2	No	No	-1.61	-0.016
SP100	P23497	>sp|P2349	MAGGGGDL	77.82	IDP	0.9971	Yes	0.9866	Yes	+		7	Driver	16	-34.3	No	No	-1.13	0.022

DDX21	Q9NR30	>sp|Q9NR	MPGKLRSDA	45.34	IDP	0.4512	No	0.9921	Yes			4	Client	22	-21.8	No	No	-0.76	0.042
DDX3X	O00571	>sp|O005	MSHVAVENA	40.94	IDP	0.8355	Yes	0.998	Yes	+		2	Driver	18	-17.3	No	No	-0.63	0.008
DDX3Y	O15523	>sp|O155	MSHVVVKN	41.21	IDP	0.8518	Yes	0.9453	Yes	+		3	Driver	18	-17.2	No	No	-0.64	0.011
FAM208A	Q9UK61	>sp|Q9UK	MATAVETEA	49.94	IDP	0.9916	Yes	0.7529	Yes	+		16	Driver	44	-15.1	No	No	-0.64	-0.015
HSPA8	P11142	>sp|P1114	MSKGPAVG	38.70	IDP	0.3211	No	0.5824	Yes			2	Client	17	-13.8	No	No	-0.46	-0.015
LMNA	P02545	>sp|P0254	METPSQRRA	87.65	IDP	0.9517	Yes	0.9161	Yes	+		6	Driver	8	-31.5	No	No	-0.86	0.005
ZC3H11A	O75152	>sp|O751	MPNQGEDC	87.53	IDP	0.9982	Yes	0.8823	Yes	+		10	Driver	16	-23.5	No	No	-0.75	0.020
PRPF38B	Q5VTL8	>sp|Q5VT	MANNSPALT	69.60	IDP	0.9991	Yes	0.9106	Yes	+		3	Driver	6	-51.9	No	No	-1.70	0.127
RBM23	Q86U06	>sp|Q86U	MASDDFDIV	65.83	IDP	0.681	Yes	0.0835	No			1	Driver	14	-23.7	No	No	-0.72	0.051
RBMX2	Q9Y388	>sp|Q9Y3	MNPLTKVKL	67.70	IDP	0.9958	Yes	0.9853	Yes	+		1	Driver	5	-45.1	No	No	-1.53	0.096
RPS27A	P62979	>sp|P6297	MQIFVKTLT	57.05	IDP	0.1763	No	0.0069	No		+	1	Client	7	-19.2	No	No	-0.84	0.115
UBA52	P62987	>sp|P6298	MQIFVKTLT	39.84	IDP	0.1393	No	0.0758	No		+	1	Client	4	-17.5	No	No	-0.70	0.121
UBB	P0CG47	>sp|P0CG	MQIFVKTLT	30.57	IDP	0.1455	No	0.0047	No		+	0	No	4	-12.2	No	No	-0.48	0.007
UBC	P0CG48	>sp|P0CG	MQIFVKTLT	28.76	Partial IDP	0.1563	No	0.0106	No		+	0	No	10	-12.3	No	No	-0.48	0.007
SRP14	P37108	>sp|P3710	MVLLESEQF	74.26	IDP	0.5059	No	0.1114	No		+	1	Client	5	-16.8	No	No	-0.44	0.110
SUB1	P53999	>sp|P5399	MPKSKELVS	86.61	IDP	0.9209	Yes	0.8756	Yes	+		1	Driver	2	-32.1	No	No	-1.09	0.055
DDX1	Q92499	>sp|Q924	MAAFSEMG	16.08	Partial IDP	0.2648	No	0.802	Yes			2	Client	23	-8.8	No	No	-0.40	0.013
FUBP1	Q96AE4	>sp|Q96A	MADYSTVPF	84.47	IDP	0.9962	Yes	0.9922	Yes	+		11	Driver	10	-24.9	No	No	-0.74	0.009
HIST1H1C	P16403	>sp|P1640	MSETAPAAP	97.65	IDP	0.9966	Yes	0.9886	Yes	+		2	Driver	3	-25.6	No	No	-0.68	0.258
HNRNPA0	Q13151	>sp|Q131	MENSQLCKL	61.97	IDP	0.9818	Yes	0.9959	Yes	+		1	Driver	5	-20.5	No	No	-0.59	0.043
HSPA1A	P31948	>sp|P3194	MEQVNELKE	55.80	IDP	0.4991	No	0.0334	No		+	5	Client	5	-26.6	No	No	-0.94	0.003
HSPA1B	V9GZ37	>tr|V9GZ3	MAKAAAIGI	37.18	IDP	0.4139	No	0.4516	No		+	2	Client	13	-14.6	No	No	-0.41	-0.017
HSPA1L	P34931	>sp|P3493	MATAKGIAI	28.39	Partial IDP	0.2245	No	0.1552	No		+	1	Client	21	-10.1	No	No	-0.34	-0.006
HSPA2	P54652	>sp|P5465	MSARGPAIG	38.03	IDP	0.3147	No	0.4757	No		+	1	Client	18	-15.1	No	No	-0.49	-0.010
HSPA5	P11021	>sp|P1102	MKLSLVAAN	41.28	IDP	0.4337	No	0.3872	No		+	3	Client	18	-14.8	No	No	-0.49	-0.029
KIAA1429	Q69YN4	>sp|Q69Y	MAVDSAME	41.78	IDP	0.9241	Yes	0.7061	Yes	+		9	Driver	57	-10.4	No	No	-0.40	-0.041
PPIAL4C	A0A0B4	>tr|A0A0E	MTPVHFSFS	10.20	Partial IDP	0.1476	No	0.0681	No		+	0	No	4	30.8	Yes	No	0.89	0.010
PPIG	Q13427	>sp|Q134	MGIKVQRPF	83.95	IDP	0.9974	Yes	0.9977	Yes	+		6	Driver	7	-57.3	No	No	-1.88	0.135
PRPF4B	Q13523	>sp|Q135	MAAAETQSL	66.63	IDP	0.9961	Yes	0.9923	Yes	+		3	Driver	15	-37.8	No	No	-1.29	0.116
PTBP1	P26599	>sp|P2659	MDGIVPDIA	43.27	IDP	0.5386	No	0.9415	Yes			4	Client	25	-2.7	No	No	-0.12	0.030
PTBP3	O95758	>sp|O957	MDGVVTDL	39.31	IDP	0.8663	Yes	0.7874	Yes	+		5	Driver	22	0.1	Yes	No	-0.10	0.030
PURB	Q96QR8	>sp|Q96Q	MADGDSGS	62.18	IDP	0.9803	Yes	0.5841	Yes	+		5	Driver	6	-24.6	No	No	-0.67	-0.014
RBM14	Q96PK6	>sp|Q96P	MKIFVGNVD	81.32	IDP	0.9999	Yes	0.9903	Yes	+		6	Driver	7	-15.4	No	No	-0.45	0.043
RBM15B	Q8NDT2	>sp|Q8ND	MKRQSERDS	61.24	IDP	0.9935	Yes	0.7781	Yes	+		5	Driver	16	-27.8	No	No	-0.85	0.058
RNF20	Q5VTR2	>sp|Q5VT	MSGIGNKRA	77.85	IDP	0.7779	Yes	0.5943	Yes	+		7	Driver	10	-34.3	No	No	-1.04	-0.011
SNRNP27	Q8WVK2	>sp|Q8WV	MGRSRSRSP	83.87	IDP	0.9856	Yes	0.8507	Yes	+		1	Driver	3	-59.2	No	No	-2.00	0.194
TOP1	P11387	>sp|P1138	MSGDHLHN	60.52	IDP	0.3929	No	0.2234	No		+	3	Client	10	-34.5	No	No	-1.29	0.060
